# NeuproGemp, a polyphenol-rich botanical formula, ameliorates Alzheimer’s-like pathology in APP/PS1 mice via inhibition of human glutaminyl cyclase

**DOI:** 10.3389/fphar.2025.1673532

**Published:** 2025-10-21

**Authors:** Tien-Sheng Tseng, Chia-Ching Liaw, Young-Ji Shiao, Ya-I Huang, Yu-Hsiu Cheng, Wang-Chuan Chen, Keng-Chang Tsai

**Affiliations:** ^1^ Institute of Molecular Biology, National Chung Hsing University, Taichung, Taiwan; ^2^ Doctoral Program in Microbial Genomics, National Chung Hsing University and Academia Sinica, Taipei, Taiwan; ^3^ National Research Institute of Chinese Medicine, Ministry of Health and Welfare, Taipei, Taiwan; ^4^ Department of Research and Development, Likang Biotechnical Co., Ltd., Tainan, Taiwan; ^5^ The School of Chinese Medicine for Post Baccalaureate, I-Shou University, Kaohsiung, Taiwan; ^6^ Department of Chinese Medicine, E-Da Hospital, Kaohsiung, Taiwan; ^7^ Ph.D. Program in Medical Biotechnology, College of Medical Science and Technology, Taipei Medical University, Taipei, Taiwan

**Keywords:** pentagalloylglucose, glutaminyl cyclase, traditional Chinese medicine, amyloid-beta, Alzheimer’s disease, zinc-dependent inhibition

## Abstract

Alzheimer’s disease (AD) is a progressive neurodegenerative disorder characterized by cognitive decline and amyloid-β (Aβ) accumulation. Increasing evidence suggests that dietary bioactive compounds may modulate neurodegenerative processes. Here, we evaluated the neuroprotective potential of NeuproGemp, a traditional Chinese functional food formula composed of *Gastrodia elata*, *Paeoniae Radix Rubra*, and the immunomodulatory protein GMI from *Ganoderma microsporum*, in APP/PS1 transgenic mice. Oral supplementation (300 mg/kg/day, 6–8 weeks) significantly improved ethological behaviors, including a ∼150% enhancement in burrowing performance (150 ± 25 g vs. 60 ± 40 g in controls), and reduced escape latency in the Morris water maze (Day 4: p < 0.05; Day 6: p < 0.01). Histological analyses demonstrated attenuated plaque-associated gliosis, with microglial/astroglial clusters reduced from 95 ± 22 to 55 ± 11 per section (p < 0.01), alongside increased hippocampal neurogenesis (DCX + cells: 49 vs. 18 cells/mm, p < 0.001). ELISA revealed reductions of ∼30% in soluble Aβ_1-42_ and ∼50% in pyroglutamate-modified Aβ_3-42_ (pE-Aβ_3-42_). High-performance liquid chromatography identified pentagalloylglucose (PGG) as the principal polyphenolic constituent of *Paeoniae Radix Rubra*, which exhibited potent human glutaminyl cyclase (hQC) inhibition (IC_50_ = 0.09 μM; KD = 63.7 nM). Molecular modeling and dynamics simulations further supported stable binding interactions of PGG and tannic acid with hQC. Collectively, these findings indicate that NeuproGemp, enriched in neuroactive polyphenols, exerts multi-targeted modulation of amyloidogenic pathways and represents a promising botanical intervention for mitigating AD-related neuropathology.

## 1 Introduction

As global populations continue to age, the incidence of neurodegenerative disorders such as Alzheimer’s disease (AD) has risen substantially (Jiang et al., 2025), underscoring the urgent need for novel therapeutic approaches that both preserve neuronal integrity and directly target disease-specific pathological mechanisms. AD, the most prevalent cause of dementia worldwide (Amiri et al., 2025), is a multifactorial neurodegenerative condition characterized clinically by progressive memory loss, cognitive impairment, and behavioral disturbances, and neuropathologically by extracellular deposition of amyloid-β (Aβ) plaques and intracellular neurofibrillary tangles (NFTs) composed of hyperphosphorylated tau protein (DeTure and Dickson, 2019; Liu et al., 2024). Among the various Aβ isoforms, Aβ_1-42_ has received particular attention due to its high aggregation propensity and neurotoxicity, acting as a central initiator of amyloidogenic cascades (Morimoto et al., 2002; Urbanc et al., 2011; Silvers et al., 2017; Haessler et al., 2024). Moreover, pyroglutamate-modified Aβ_3-42_ (pE-Aβ_3-42_), a truncated and post-translationally modified variant generated through the enzymatic activity of glutaminyl cyclase (QC) (Becker et al., 2013; Bridel et al., 2017), has emerged as a highly pathogenic Aβ species. This variant is resistant to proteolytic degradation, aggregates rapidly, and exhibits significantly increased neurotoxicity compared to unmodified Aβ peptides (Busby et al., 1987; Fischer and Spiess, 1987; Pohl et al., 1991; Van Coillie et al., 1998).

QC (EC 2.3.2.5), first discovered in Carica papaya in 1946, is a zinc-dependent acyl transferase widely conserved across species (Busby et al., 1987; Fischer and Spiess, 1987; Pohl et al., 1991; Song et al., 1994; Bockers et al., 1995; Fraser, 1998; Dahl et al., 2000; Schilling et al., 2002). In mammals, human QC (hQC) is highly expressed in the brain—particularly in the hippocampus and hypothalamus—as well as in peripheral endocrine tissues, where it modulates the maturation and stability of neuropeptides and hormones (Busby et al., 1987; Fischer and Spiess, 1987; Pohl et al., 1991). Structurally, hQC is distinct from other zinc metalloproteins due to its mono-zinc coordination, conferring a unique catalytic mechanism (Schilling et al., 2003; Huang et al., 2005a; Huang et al., 2005b). Recent studies have implicated hQC in the pathogenesis of AD, with increased QC activity closely associated with the accumulation of pE-Aβ_3-42_ in cerebral plaques and blood vessels (Saido et al., 1996; Harigaya et al., 2000; Ezura et al., 2004; Batliwalla et al., 2005; Muthusamy et al., 2006; Thouennon et al., 2007; Cynis et al., 2008; Schilling et al., 2008a; Schilling et al., 2008b; Thal et al., 2015). Animal models further validate this link: pE-Aβ_3-42_ not only exacerbates the neurotoxicity of Aβ_1-42_ but also contributes to behavioral deficits, whereas genetic ablation of hQC in 5xFAD transgenic mice reduces both plaque burden and cognitive decline (Soto and Estrada, 2008; Wirths et al., 2009; Jawhar et al., 2011b). Beyond neurodegeneration, aberrant hQC expression has been reported in peripheral immune and tumorigenic contexts, including monocyte-mediated inflammation in rheumatoid arthritis and immunomodulation in melanoma (Batliwalla et al., 2005; Gillis, 2006; Huang et al., 2011). Accordingly, hQC represents a promising therapeutic target. Several hQC inhibitors developed by pharmaceutical entities such as Probiodrug AG have demonstrated preclinical efficacy, with PQ912 advancing to Phase I clinical trials (Schilling et al., 2008b; Demuth et al., 2010; Jawhar et al., 2011a).

Given that over 40 million individuals globally are affected by AD and that current treatment modalities offer limited efficacy (Dobson, 2015), targeting hQC to reduce neurotoxic pE-Aβ accumulation is a compelling strategy to mitigate disease progression. Recently, immunotherapeutic approaches have aimed to remove pathological Aβ conformers via monoclonal antibodies with varying specificity. Aducanumab binds aggregated Aβ (oligomers and fibrils) and promotes microglial clearance, though its clinical benefits remain controversial due to mixed cognitive outcomes and amyloid-related imaging abnormalities (ARIA) (Haddad et al., 2022; Shi et al., 2022; Vaz et al., 2022). Lecanemab selectively targets soluble protofibrillar Aβ, preventing fibril formation and demonstrating modest cognitive improvements (Shi et al., 2022; Vitek et al., 2023). Donanemab, notably, is designed to recognize pE-Aβ_3-42_ species within mature amyloid plaques, offering specificity toward this highly pathogenic Aβ form (Rashad et al., 2022; Terao and Kodama, 2024b). Early trials show encouraging plaque clearance and potential cognitive benefits, positioning it as a strategic tool against pyroglutamate-Aβ–driven pathology (Terao and Kodama, 2024a; 2025). Together, these antibodies exemplify a conformer-specific therapeutic paradigm in AD: Aducanumab targets aggregated Aβ, Lecanemab targets protofibrils, and Donanemab targets pE-Aβ plaques.

Despite these advances, current amyloid-targeting therapies remain primarily preventative or focused on clearing amyloid deposits, with limited capacity to repair neuronal damage that has already occurred. In this context, Traditional Chinese Medicine (TCM) provides a complementary approach, having long employed herbal formulations to preserve cognitive function, support neuroprotection, and promote neuroregeneration. Among the most extensively studied herbs, *Panax ginseng* (Ren Shen) and *Ginkgo biloba* (Yinxing) demonstrate neuroprotective effects by modulating neurotransmission and reducing oxidative stress, thereby helping to mitigate cognitive decline (Oken et al., 1998; Kennedy et al., 2001; 2002; Snitz et al., 2009; Wan et al., 2015; Park et al., 2019; Li et al., 2020; Li et al., 2023). P*olygala tenuifolia* (Yuan Zhi) enhances neurogenesis and suppresses inflammation, processes essential for memory consolidation (Wang et al., 2019; Wang et al., 2021; Chen et al., 2022). The classical formula Si Wu Tang, which contains *Angelica sinensis* and *Rehmannia glutinosa*, exhibits antioxidative and anti-inflammatory properties (Cheng et al., 2021; Fu et al., 2022; Zhao et al., 2024). Additionally, *Astragalus membranaceus* contribute neuroprotection through antioxidation, inflammation suppression, and mitochondrial support (Niu et al., 2004; Liu et al., 2006; Shuchang et al., 2008; Tsai et al., 2011; Huang et al., 2017; Liu et al., 2018; Chen et al., 2020; Tan et al., 2020; Calonghi et al., 2021; Fasina et al., 2022; Huang et al., 2022; Chen F. et al., 2023; Meng et al., 2023; Fan et al., 2024; Li et al., 2024; Shi and Ma, 2024). Although many TCM herbs show neuroprotective or cognitive benefits, their direct effects on key Alzheimer’s disease (AD) pathologies—especially inhibiting Aβ_1-42_ aggregation and suppressing pyroglutamate-modified Aβ_3-42_ (pE-Aβ_3-42_) formation—are not well understood. While reducing Aβ_3-42_ deposition may limit neurotoxicity, it cannot reverse existing neuronal loss. Thus, discovering herbs that both inhibit Aβ aggregation and promote neuronal regeneration is crucial. Ideal therapies would block neurotoxic cascades and simultaneously stimulate neurogenesis, restore synaptic connections, and repair damaged circuits. With this dual focus, TCM represents a valuable yet underexplored source for developing multitargeted AD treatments. Further research on how specific herbs modulate Aβ species, glutaminyl cyclase (QC) activity, and regenerative pathways is needed to fully unlock their therapeutic potential.

Despite growing recognition of human glutaminyl cyclase (hQC) as a promising therapeutic target for Alzheimer’s disease (AD), research has primarily focused on synthetic inhibitors, while natural compounds with potentially safer and multitargeted effects remain largely unexplored. In particular, whether polyphenols such as pentagalloylglucose (PGG) and tannic acid can inhibit hQC activity and attenuate AD-related pathology has not been systematically evaluated. To address this gap, the present study investigated the therapeutic potential of NeuproGemp, a traditional Chinese medicine formulation comprising *Gastrodia elata*, *Paeoniae Radix Rubra*, and *Ganoderma microsporum* immunomodulatory protein (GMI), in APP/PS1 mice. We aimed to determine whether NeuproGemp and its bioactive constituents modulate hQC activity, reduce Aβ deposition and neuroinflammation, and improve cognitive function. Our findings designate NeuproGemp as a validated multifunctional therapeutic candidate for AD, demonstrating that its neuroprotective efficacy is significantly mediated through the inhibition of hQC, a pivotal enzyme in the generation of the neurotoxic pyroglutamate-modified Aβ_3-42_ (pE-Aβ_3-42_). Chronic administration in APP/PS1 transgenic mice led to substantial reductions in cortical and hippocampal levels of Aβ_1-42_ and pE-Aβ_3-42_, revealing marked attenuation of amyloid pathology *in vivo*. Through biochemical assays and molecular docking, we identified PGG as the primary active compound, with a potent IC_50_ of 0.09 µM, while tannic acid exhibited complementary inhibitory activity (IC_50_ = 0.36 µM). Beyond hQC inhibition, both compounds displayed multifaceted biological properties—including antioxidative, anti-inflammatory, and metabolic regulatory effects—that collectively contribute to the multitargeted neuroprotective profile of NeuproGemp. These results not only bridge the conceptual gap between traditional botanical remedies and contemporary molecular pharmacology but also underscore the translational potential of NeuproGemp as a holistic, mechanism-based intervention for AD, with future studies warranted to optimize clinical formulation, dosing strategies, and long-term safety evaluation to advance its development toward therapeutic application in human neurodegenerative diseases.

## 2 Materials and methods

### 2.1 Plant material

Five traditional Chinese Medicines, including *Paeoniae Radix alba* (*Paeonia lactiflora* Pall, PA), *Paeoniae Radix Rubra* (*P. lactiflora* Pall, PR), *Gastrodiae Rhizoma* (*G. elata* Blume, GE), *Chebulae Fructus* (*Terminalia chebula* Retz., TC), and GMI (*G. microsporum* immunomodulatory protein), were provided by Li Kang Biotechnical Co., Ltd. In Taiwan.

### 2.2 Animals, housing and behavioral assessments

Male APPswe/PS1ΔE9 transgenic mice (Jackson Laboratory, No. 005864) expressing Swedish mutant APP695 and PS1 lacking exon 9 were used, along with their wild-type littermates (Jankowsky et al., 2004). Mice were housed at 24 °C ± 1 °C and 55%–65% humidity under a 12:12 h light–dark cycle (07:00–19:00). All procedures were approved by the Institutional Animal Care and Use Committee at the National Research Institute of Chinese Medicine (Approval Nos. 112-417-1 and 113-417-2). Sample size was determined by G*Power analysis, which indicated n = 17 per group for a large effect (d = 1.0); in accordance with the 3R principle, n was set between 5 and 12 per group.) To evaluate the effects of NeuproGemp on behavioral deficits in APP/PS1 mice, we employed burrowing, Morris water maze (MWM), and novel object recognition (NOR) tests. Burrowing is a species-specific, multi-brain-dependent spontaneous behavior (Deacon, 2006) and reflects activities of daily living (ADL), which are impaired in Alzheimer’s disease and in APP/PS1 mice (Jirkof, 2014; Woodling et al., 2016; Tzeng et al., 2018). The MWM assesses hippocampal-dependent spatial learning and memory by motivating mice to locate a hidden platform using extra-maze cues (Brandeis et al., 1989; Vorhees and Williams, 2006; Nunez, 2008). The NOR test evaluates recognition memory based on rodents’ natural preference for novelty, requiring minimal training or reinforcement (Lueptow, 2017). Together, these assays provide complementary and clinically relevant measures of cognitive and functional deficits in this AD model.)

### 2.3 Burrowing and novel object recognition tests

Burrowing behavior was assessed in APP/PS1 transgenic mice after 6 weeks of oral administration with NeuproGemp (300 mg/kg/day) or vehicle, as previously described (Tzeng et al., 2018; Hsu et al., 2020) with minor modifications. Briefly, mice were placed individually into polysulfone cages (42.5 × 26.6 × 18.5 cm) containing a burrowing tube (20 cm long, 7 cm diameter), elevated 2–2.5 cm at the open end. Each tube was filled with 230 g of standard mouse food pellets. After 2 h, the remaining food in the tube was weighed to calculate the amount displaced (burrowed) by each mouse. Novel object recognition (NOR) was conducted 1–2 days after the burrowing task to evaluate recognition memory. The test consisted of three phases: habituation, training, and testing. During habituation, mice were allowed to freely explore an empty open-field arena (40 × 40 × 40 cm) for 10 min. On the following day, two identical objects were placed in opposite corners of the arena, and mice were allowed to explore for 10 min (training phase). After a 1-h retention interval, one of the familiar objects was replaced with a novel object of similar size, and mice were again allowed to explore for 10 min (testing phase). The discrimination index was calculated as the time spent exploring the novel object divided by the total time exploring both objects.

### 2.4 Morris water maze test

Spatial learning and memory of APP/PS1 transgenic mice were evaluated using the Morris water maze (MWM) after 6 weeks of oral NeuproGemp administration (300 mg/kg/day). The test was conducted from day 49–64, based on a modified protocol from (Tzeng et al., 2018). The apparatus consisted of a circular pool (120 cm diameter, 40 cm height) filled with opaque water at 25 °C ± 1 °C to a depth of 20 cm. A hidden platform (10 cm diameter) was submerged 1 cm below the surface in one quadrant, which remained constant throughout testing. Mice underwent six trials per day for six consecutive days. Each trial lasted up to 60 s; if the platform was found, the mouse remained on it for 30 s. If not, it was guided to the platform and allowed to stay for the same duration. Inter-trial intervals were set at 60 s. Escape latency (time to reach the platform) was recorded. Representative swim paths were documented on the first and sixth trials. On day 4 and 6, NeuproGemp-treated mice (n = 12) showed significantly reduced escape latencies compared to the vehicle group (n = 6), indicating enhanced spatial learning.

### 2.5 Immunohistochemistry

Immunohistochemistry was performed to evaluate amyloid plaque deposition and associated glial clustering in the brain of APP/PS1 transgenic mice following NeuproGemp treatment. 5-month-old APP/PS1 mice were orally administered with vehicle, HE-A (30 mg/kg/day), or NeuproGemp (300 mg/kg/day) for 8 weeks. After treatment, mice were sacrificed and brains were harvested and sectioned coronally at 30 µm using a cryostat. Free-floating brain sections were washed in phosphate-buffered saline (PBS) and blocked for 1 h in PBS containing 1% bovine serum albumin (BSA), 3% normal donkey serum, and 0.3% Triton X-100. Sections were then incubated overnight at 4 °C with the following primary antibodies diluted in blocking buffer: mouse monoclonal anti-Aβ_1_–_16_ antibody (AB10, Millipore, MAB5208) to detect amyloid plaques (red), goat polyclonal anti-Iba-1 antibody (Abcam, ab5076) for microglia (green), and mouse monoclonal anti-GFAP antibody (Millipore, MAB5804) for reactive astrocytes (blue). Following primary incubation, sections were rinsed and incubated at room temperature for 2 h with the corresponding secondary antibodies: RRX-conjugated donkey anti-mouse IgG, Alexa Fluor 647-conjugated donkey anti-goat IgG, and Hoechst 33258 (2 μg/mL, Invitrogen) for nuclear staining. All secondary antibodies were purchased from Jackson ImmunoResearch (West Grove, PA, United States). After washing in PBS containing 0.01% Triton X-100, sections were mounted with Aqua Poly/Mount (Polysciences Inc.) and imaged using a Zeiss LSM 780 confocal microscope (Jena, Germany). Representative confocal images were acquired with a 10-μm z-stack and projected using maximal intensity projection. The number of plaque-associated microglial and astroglial clusters in the parietal cortical and hemispheric sections was quantified using ImageJ software (NIH, Bethesda, MD, United States of America). Results were analyzed using Student’s t-test, and data are presented as mean ± SD.

### 2.6 Detection of hippocampal neurogenesis

To assess hippocampal neurogenesis, 5-month-old APP/PS1 transgenic mice were orally administered with either vehicle or NeuproGemp (300 mg/kg/day) for 8 weeks. After treatment, brain sections were processed for immunohistochemical staining. Antigen retrieval was performed by incubating free-floating sections in 10 mM sodium citrate buffer (pH 6.0) at 80 °C for 30 min, followed by treatment with 2 M HCl at 37 °C for 30 min to denature DNA for BrdU detection. Sections were then incubated in blocking buffer (PBS containing 1% BSA, 3% normal donkey serum, and 0.3% Triton X-100) for 1 h, followed by incubation with primary antibodies at 4 °C overnight. The primary antibodies used included mouse monoclonal anti-BrdU antibody (Santa Cruz Biotechnology, sc-32323) and rabbit polyclonal anti-doublecortin (DCX) antibody (Abcam, ab18723). After washing, sections were incubated with fluorescently labeled secondary antibodies as described previously, along with Hoechst 33258 for nuclear staining. Images of the dentate gyrus were acquired using a Zeiss LSM 780 confocal microscope. DCX-positive (red) and BrdU-positive (green) cells within the subgranular zone (SGZ) of the dentate gyrus were quantified. The linear density of labeled cells was calculated as the number of positive cells per millimeter of SGZ. To further evaluate neuronal differentiation and dendritic development, the dendritic complexity of DCX-positive immature neurons in the dentate gyrus was analyzed by laminar quantification of disjointed dendritic processes. The number of DCX-positive cell bodies (a), primary dendrites (b), secondary dendrites (c), and tertiary dendrites (d) was counted. Dendritic branching complexity was assessed by calculating the ratios b/a, c/b, and d/c to represent the level of primary dendrite sprouting and the extent of secondary and tertiary dendritic arborization. Statistical comparisons between vehicle- and NeuproGemp-treated groups were performed using Student’s t-test. Data are presented as mean ± SD.

### 2.7 HPLC analysis

Each of *Paeoniae R. alba* (0.5 g, powder), *Paeoniae Radix Rubra* (0.5 g, powder), *Gastrodiae Rhizoma* (0.5 g, powder), and *Chebulae Fructus* (0.1 g, powder) were extracted with 50% methanol (20 mL) by ultrasonicate for 30 min. After filtered through Whatman No. 1 paper, the filtrates put into a 20 mL volumetric flask containing 50% methanol. The filtrate was further filtered additionally through a 0.22 μm membrane for analysis. The reference compound, pentagalloylglucose (PGG, Sigma-Aldrich, CAS: 14937-32-7) was accurately weighed and dissolved in 50% methanol to obtain stock solutions at 1.0 mg/mL. The stock solutions were then diluted to get different concentrations in the range of 0.12–242.55 μg/mL for PGG. The HPLC profile was performed on a Shimadzu Nexera-*i* LC-2060C 3D Liquid Chromatograph (Shimadzu, Kyoto, Japan) equipped with an ODS COSMOSIL 5C_18_-AR-II column (4.6 mm × 250 mm) at 35 °C. In HPLC fingerprint analysis for PGG, the mobile phase consisted of D.D water with 0.1% phosphoric acid (A) and acetonitrile with 0.1% phosphoric acid (B) using a gradient condition as follows: 0.01–7.00 min, 5%–10% B; 7−10 min, 10%–15% B; 10–22 min, 15%–18% B; 22–40 min, 18%–20% B; 40–45 min, 20%–100% B, and 45–55 min, 100%–100% B. In HPLC fingerprint analysis for TCA, the mobile phase consisted of 10 mM potassium dihydrogen phosphate buffer (A) and acetonitrile/methanol 20:80 (B) using isotopic condition (25% B and 10 min). The mobile phase flow rate and the injection volume were 1.0 mL/min and 1-10 μL (the standard compound: 1 μL; GE filtrate: 40 μL; PA and PR filtrate: 10 μL; TC filtrate: 40 μL), respectively. The detection wavelength was 280 nm. The UV spectra were recorded between 190 nm and 800 nm. Furthermore, the sample filtrates were injected into the HPLC-DAD system and immediately analyzed to quantity the reference compounds. The contents of the markers were calculated based on the corresponding calibration curve.

### 2.8 Expression and purification of hQC

The coding sequence of human glutaminyl cyclase (hQC), covering residues Ala33 to Leu361, was cloned into the pQE-80L expression vector utilizing the restriction sites BamHI and HindIII. This recombinant plasmid was introduced into *E. coli* BL21 (DE3) cells through heat shock transformation. For protein expression, the transformed *E. coli* cells were grown in Luria-Bertani (LB) medium supplemented with 100 mg/L ampicillin at 37 °C. Once the optical density at 600 nm (OD_600_) reached 0.6, protein expression was induced using 0.1 mM isopropyl β-D-1-thiogalactopyranoside (IPTG), and the culture was incubated at 24 °C for 48 h, optimizing for soluble protein yield under reduced temperature conditions. After induction, the cells were harvested by centrifugation at 6,000 rpm for 20 min. The resulting cell pellet was resuspended in a lysis buffer containing 50 mM Tris-HCl (pH 8.5), 150 mM NaCl, and 20 mM imidazole. Cell disruption was performed using high-pressure homogenization via a microfluidizer (Microfluidics). The lysate was clarified through centrifugation, and the soluble protein fraction was purified using nickel-nitrilotriacetic acid (Ni-NTA) affinity chromatography, leveraging the polyhistidine tag for selective binding to the resin. The bound hQC protein was eluted and further purified using standard chromatographic techniques. The purity of the isolated hQC was confirmed by SDS-PAGE, followed by Coomassie Brilliant Blue staining, demonstrating a purity level exceeding 95%. This level of purity is crucial for conducting subsequent structural and functional analyses of the hQC protein.

### 2.9 hQC inhibition assay

The inhibitory effects of various compounds on human glutaminyl cyclase (hQC) were assessed using a validated spectrophotometric assay. In this assay, glutamic dehydrogenase (EC 1.4.1.3; Sigma-Aldrich) was utilized as a coupling enzyme to indirectly quantify hQC activity, employing H-Gln-Gln-OH (Bachem) as the specific substrate. The reaction mixture consisted of 50 mM Tris-HCl (pH 8.0), 0.25 mM NADH, 30 U of glutamic dehydrogenase, 15 mM α-ketoglutaric acid, and 0.1 μM hQC. Inhibitors were introduced to the reaction mixture and allowed to incubate for 5 min at 25 °C to promote interaction with the enzyme. The enzymatic reaction was initiated by adding 1 mM of the substrate, and the progression was monitored by measuring the absorbance at 340 nm, corresponding to the oxidation of NADH. The IC_50_ values for the inhibitors were calculated by plotting initial reaction rates against the logarithmic concentrations of the inhibitors. Nonlinear regression fitting was conducted using GraphPad Prism 6 software, enabling accurate quantification of the inhibitors’ potencies and facilitating a detailed analysis of their inhibitory effects on hQC. This methodology highlights the critical parameters necessary for understanding the inhibitory dynamics of hQC, which are essential for developing therapeutic agents targeting this enzyme.

### 2.10 Localized surface plasmon resonance (LSPR)

The binding affinity of the inhibitor to human glutaminyl cyclase (hQC) was assessed using an OpenSPR instrument (Nicoya Lifesciences Inc.). The hQC protein solution was prepared in a Tris-T buffer consisting of 50 mM Tris-HCl (pH 7.4), 150 mM NaCl, and 0.005% Tween 20. For the immobilization process, hQC was applied at a concentration of 80 μg/mL onto an NTA sensor chip, followed by exposure to the inhibitors in the fluid phase. The inhibitor analyte solutions were formulated in Tris-T buffer containing 0.5% DMSO and 2% BSA at various concentrations. Prior to each experimental run, the sensor chip was regenerated with a 10 mM glycine-HCl buffer at pH 2.2 to ensure a consistent baseline for binding measurements. The resulting data were analyzed using a 1:1 binding model fitted with Trace Drawer software to determine the dissociation constant (KD) value, which provides insight into the binding strength between the inhibitor and hQC. This methodology is crucial for evaluating potential therapeutic agents targeting hQC by elucidating their binding characteristics.

### 2.11 Molecular modelling of hQC-inhibitor complex

Molecular modeling techniques were employed to investigate the complex structure formed by the interaction between human glutaminyl cyclase (hQC) and its inhibitors. The complex structure of hQC-PQ912 (PDB ID: 8XGB) was employed for molecular docking simulation. Before docking calculations, the binding site was delineated by identifying key interactive residues which essentially interact with PQ912, crucial for flexible docking of the protein-ligand complex. The GOLD docking program (CCDC, version 5.1) was utilized for this purpose, leveraging the GoldScore scoring function to evaluate the binding affinities of various inhibitor candidates. In the flexible docking process, the side chains of the identified binding site residues were permitted to adopt multiple rotameric configurations to better reflect the dynamic nature of the protein-ligand interactions. Prior to docking, the inhibitors underwent rigorous construction and energy minimization, ensuring their geometric configurations were optimized for interaction with hQC. Key docking parameters were carefully calibrated, including a defined number of operations (set to 1,600,000) and a population size of 1,000, while maintaining default settings for other parameters. This comprehensive approach facilitated the identification of the most probable orientation and positioning of the inhibitors within the binding site, informed by free energy considerations that suggest favorable interactions.

### 2.12 Molecular dynamic simulations

The structural conformations of PGG and tannic acid bound to hQC were obtained from molecular docking and further subjected to molecular dynamics (MD) simulations. Prior to MD simulations, the protein-ligand complex structures underwent error correction using the “Prepare Proteins” protocol in Discovery Studio 2021. For the hQC–inhibitor complexes, structural inconsistencies were similarly addressed through this protocol. The complexes were solvated in an orthorhombic simulation box using the CHARMm force field, maintaining a physiological ionic strength of 0.15 M NaCl to ensure system neutrality. The solvation process involved the addition of 8,525 water molecules, 23 sodium ions, and 23 chloride ions for the hQC control complex. Corresponding inhibitor-bound complexes were similarly solvated, with minor variations dictated by system size. Energy minimization was conducted in two steps: the first utilized 5,000 steps of the steepest descent algorithm, followed by 5,000 steps of the conjugate gradient method, optimizing the system’s initial geometries. The minimized systems were gradually heated to 300 K over 20 ps, followed by a 500-ps equilibration phase using the Standard Dynamic Cascade protocol in Discovery Studio 2021. Production MD simulations were conducted for 100 ns under the NVT ensemble at 300 K, with trajectory snapshots saved every 500 ps. Electrostatic interactions were computed using the Particle Mesh Ewald (PME) method, and the SHAKE algorithm was employed to constrain hydrogen-containing bonds, permitting a 2-fs time step. The Generalized Born implicit solvent model was implemented to enhance computational efficiency while preserving accuracy. Post-simulation analyses were performed using the “Analyze Trajectory” tool in Discovery Studio 2021. Key parameters evaluated included root mean square deviation (RMSD), root mean square fluctuation (RMSF), and radius of gyration (Rg), providing insights into the dynamic behavior and structural integrity of the hQC-inhibitor complexes. RMSD analyses quantified the overall conformational stability of the protein-ligand systems, while RMSF revealed residue-specific flexibility. The Rg values assessed structural compactness, highlighting the inhibitors’ effects on protein stability and dynamic properties throughout the simulation trajectory.

### 2.13 Management and administration

The animal protocol for this study received approval from the Institutional Animal Care and Use Committee at the National Research Institution of Chinese Medicine (IACUC No: 112-417-1). All procedures involving animals and their care adhered to the guidelines set forth in the Guide for the Care and Use of Laboratory Animals published by the United States National Institutes of Health (NIH). APP/PS1 transgenic mice were procured from Jackson Laboratory (No. 005864). APP/PS1 double-transgenic mice co-express the Swedish mutant human APP695 and a mutant form of human presenilin 1 (PS1) lacking exon 9 (Jankowsky et al., 2004). This model exhibits progressive amyloid-β (Aβ) accumulation and plaque formation beginning at 3–5 months of age (Malm et al., 2011), followed by impairments in spatial learning and memory around 6 months (Xiong et al., 2011; Tzeng et al., 2018). In addition to amyloid pathology, APP/PS1 mice display cholinergic system degeneration and behavioral deficits resembling those observed in Alzheimer’s disease patients (Malm et al., 2011). APP/PS1 mice display age-dependent hippocampal synaptic dysfunction, neuroinflammation, and cognitive deficits from mid-age onward, providing a reproducible and mechanistically tractable model to evaluate interventions targeting synaptic integrity, neuroinflammation, and memory (Bui et al., 2024; Zhong et al., 2024). These features make the APP/PS1 mouse a widely accepted and reliable model for studying AD-related mechanisms and evaluating potential therapeutic interventions.) The breeding ratio employed was one male to two females per cage. The experimental subjects included wild-type siblings and transgenic female C57BL/6J mice exhibiting Alzheimer’s disease (AD) phenotypes. The animals were maintained under controlled environmental conditions, with a room temperature of 24 °C ± 1 °C, humidity levels between 55% and 65%, and a 12-h light-dark cycle (07:00–19:00). All procedures were conducted in compliance with the NIH guidelines. The mice were fed a standard rodent diet and had free access to water. For the investigation of short-term therapeutic effects, APP/PS1 mice (Five months old; body weight, 30 g) were administered either a vehicle control (n = 6) or the NeuproGemp (300 mg/kg/day, n = 12) via oral gavage starting at 5 months of age for a duration of 8 weeks. In parallel, wild-type (WT) mice received the vehicle treatment at 5 months of age for a shorter period of 2 weeks (n = 6). At the end of the experiment, the mice were deeply anesthetized with avertin and then sacrificed by transcardial perfusion with 50 mL of saline. The brain was collected and subjected to mechanical disruption in a homogenization buffer composed of 20 mM Tris-HCl at pH7.4, 320 mM sucrose, 2 mM EDTA, 1 mM PMSF, 5 μg/mL leupeptin, and 5 μg/mL aprotinin. Aβ level were measured by human Aβ_1-42_ and N3PEAβ_1-42_ Enzyme-Link Immunosorbent Assay (ELISA) kit (Invitrogen, KHB3442 and IBL, 27716). The detail experiments were performed according to the manufacture’s protocol.

## 3 Results

### 3.1 NeuproGemp improves ethological behavior in APP/PS1 mice

**FIGURE 1:**
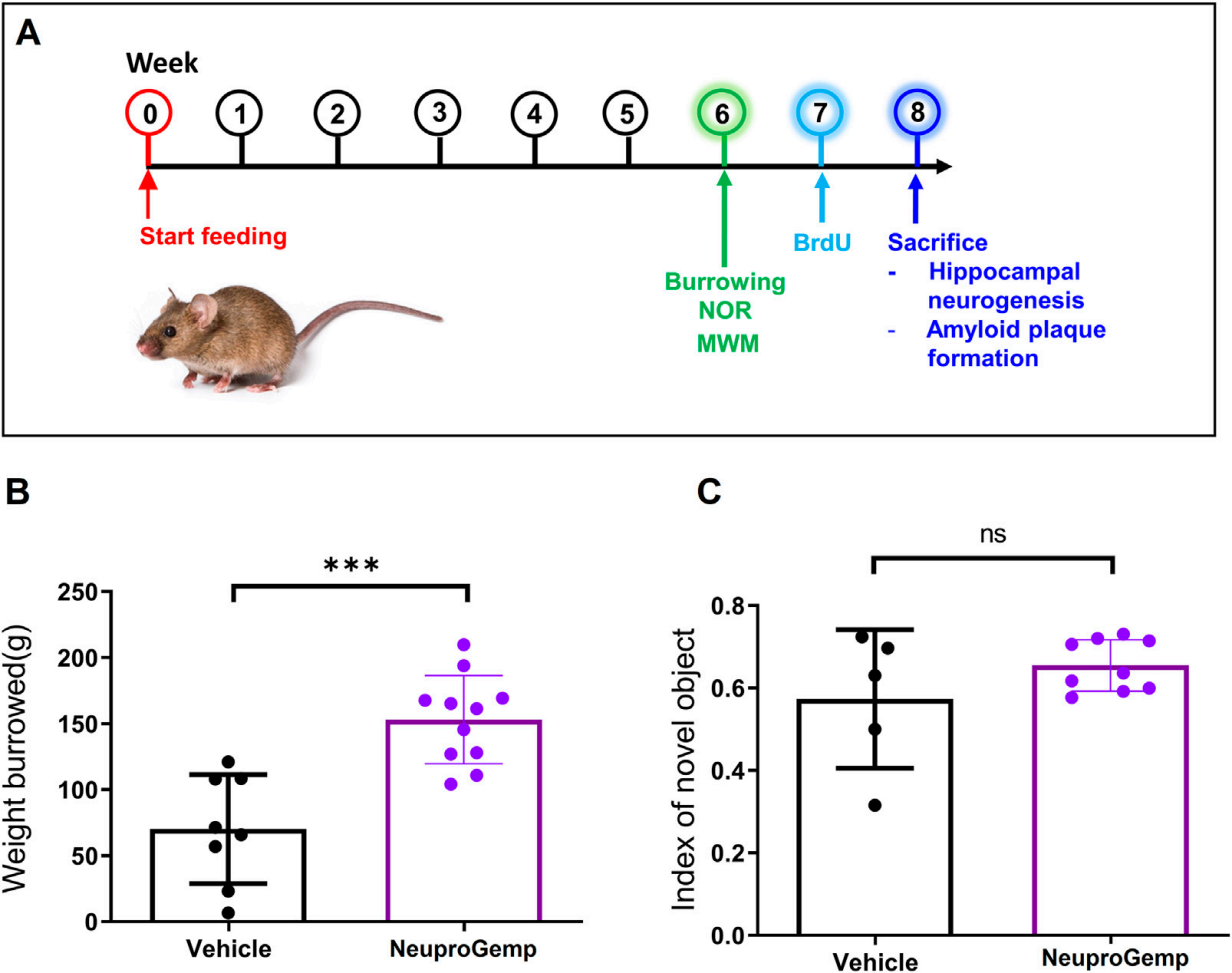
Effects of NeuproGemp on burrowing behavior and novel object recognition in APP/PS1 mice. **(A)** Experimental timeline: 5-month-old APP/PS1 transgenic mice were orally administered either vehicle or NeuproGemp (300 mg/kg/day) for 6 weeks. Burrowing and novel object recognition (NOR) tasks were conducted during the 6th week of treatment. **(B)** Burrowing task: Total amount of pellets displaced over a 2-h period. APP/PS1 mice treated with vehicle (n = 8) or NeuproGemp (n = 11) are shown. **(C)** Novel object recognition task: Discrimination index calculated as (time spent with novel object–time spent with familiar object)/total exploration time. Data are presented as mean ± SD. Statistical analysis was performed using unpaired two-tailed t-tests, with significance indicated as ***, p < 0.001. All behavioral tests were conducted under standardized lighting and noise conditions, and mice were habituated to the testing room for at least 30 min prior to assessment.

In 5-month-old APP/PS1 transgenic mice, 6 weeks of daily oral NeuproGemp treatment (300 mg/kg/day) (Figure 1A) led to a marked improvement in ethologically relevant behavior, as shown in the burrowing assay: NeuproGemp-treated mice displaced ∼150 ± 25 g of substrate compared to ∼60 ± 40 g in vehicle controls—a highly significant enhancement (Figure 1B). This ∼150% increase suggests that NeuproGemp not only restored but strongly enhanced species-specific motivated behaviors commonly impaired in Alzheimer’s models. In contrast, in the novel object recognition task, NeuproGemp-treated mice showed a modest but nonsignificant improvement in the discrimination index (0.65 ± 0.10 vs. 0.55 ± 0.15) (Figure 1C), indicating a trend toward enhanced recognition memory that did not reach statistical significance under current experimental conditions. Additionally, NeuproGemp administration did not significantly affect body weight or food intake in mice, indicating no overt systemic toxicity or impact on feeding behavior (Supplementary Figures S1, S2).

### 3.2 NeuproGemp improved the spatial learning and memory in APP/PS1 mice

**FIGURE 2:**
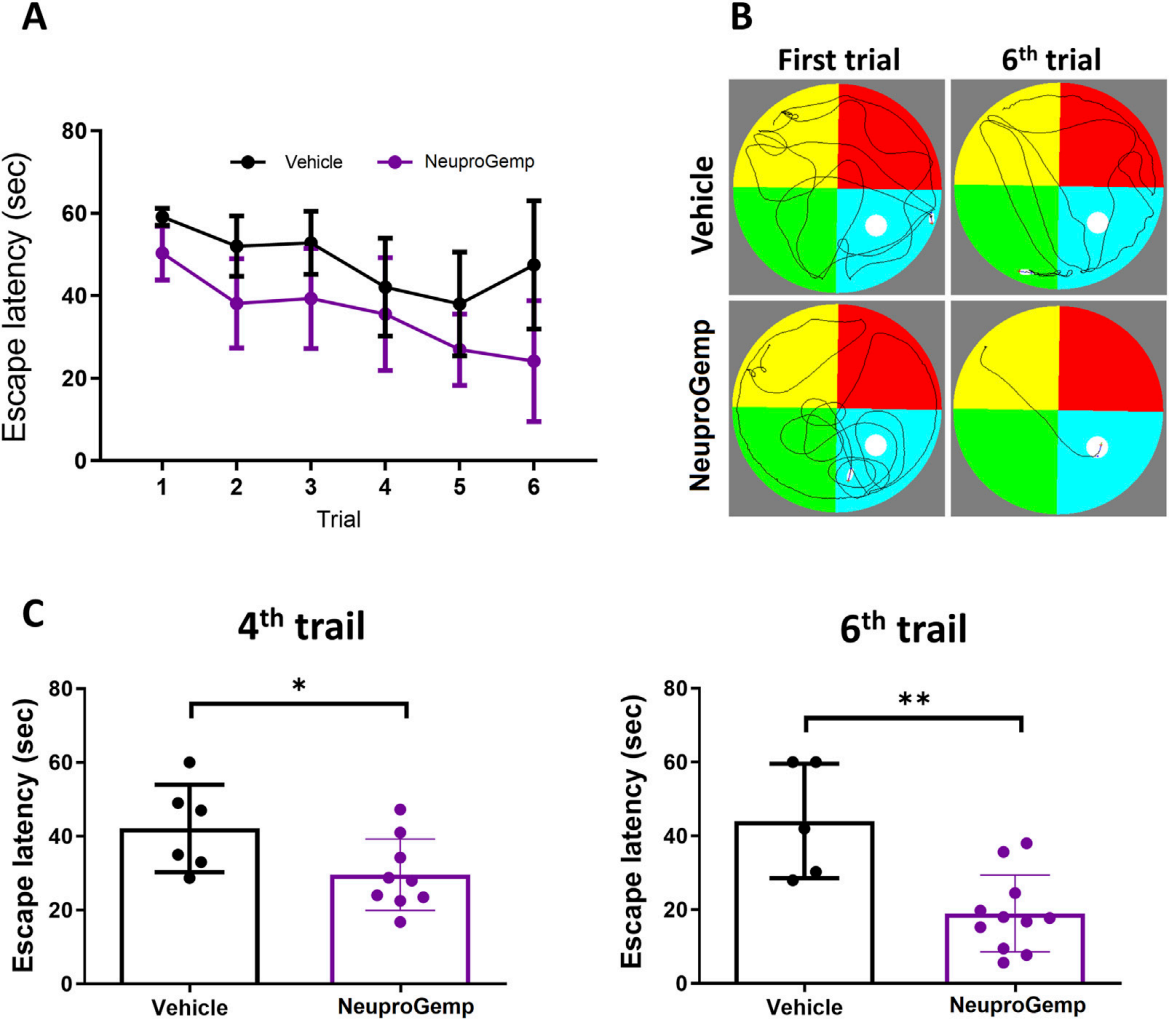
NeuproGemp improves spatial learning and memory in APP/PS1 mice. APP/PS1 transgenic mice were orally administered vehicle or NeuproGemp (300 mg/kg/day) for 6 weeks. Spatial learning and memory were assessed using the Morris water maze (MWM) during the 6th week. **(A)** Hidden platform training: Escape latency measured over six consecutive days. Data represent mean ± SD. Statistical analysis was performed using two-way ANOVA with repeated measures for training days. **(B)** Representative swim paths of APP/PS1 mice from the first and sixth trials of the hidden platform test. **(C)** Escape latency on day 4 and day 6 of hidden platform trials. APP/PS1 mice treated with NeuproGemp (n = 12) showed significantly reduced escape latency compared with vehicle-treated mice (n = 6). Data are presented as mean ± SD and analyzed using unpaired two-tailed t-tests. Significance is indicated as *, p < 0.05; **, p < 0.01. All behavioral testing was conducted under controlled lighting and noise conditions, and mice were habituated to the testing room for at least 30 min prior to assessment.

To assess the effect of NeuproGemp on spatial learning and memory in Alzheimer’s disease (AD) model mice, APP/PS1 transgenic mice aged 5 months were orally administered NeuproGemp (300 mg/kg/day) for 6 weeks. Following treatment, mice underwent the Morris water maze (MWM) test, a well-established paradigm for evaluating hippocampus-dependent spatial memory. During the 6-day acquisition phase of the hidden platform task, the NeuproGemp-treated group exhibited a progressive decline in escape latency compared to the vehicle-treated group (Figures 2A,B). Statistical analysis revealed a significant effect of treatment and training day, indicating enhanced learning across days in the NeuproGemp group. Specifically, on day 4 and day 6, escape latency was significantly shorter in the NeuproGemp group compared to vehicle controls (p < 0.05 and p < 0.01, respectively; Figure 2C), suggesting that NeuproGemp treatment facilitated more efficient spatial learning. Representative swim path diagrams illustrated that NeuproGemp-treated mice developed more direct and goal-oriented search strategies by the sixth trial, whereas vehicle-treated mice continued to display dispersed and random swimming patterns (Figure 2B). These findings indicate that NeuproGemp significantly improved spatial acquisition and memory retention in APP/PS1 mice.

### 3.3 Effect of NeuproGemp on plaque-associated clusters in APP/PS1 mice

**FIGURE 3:**
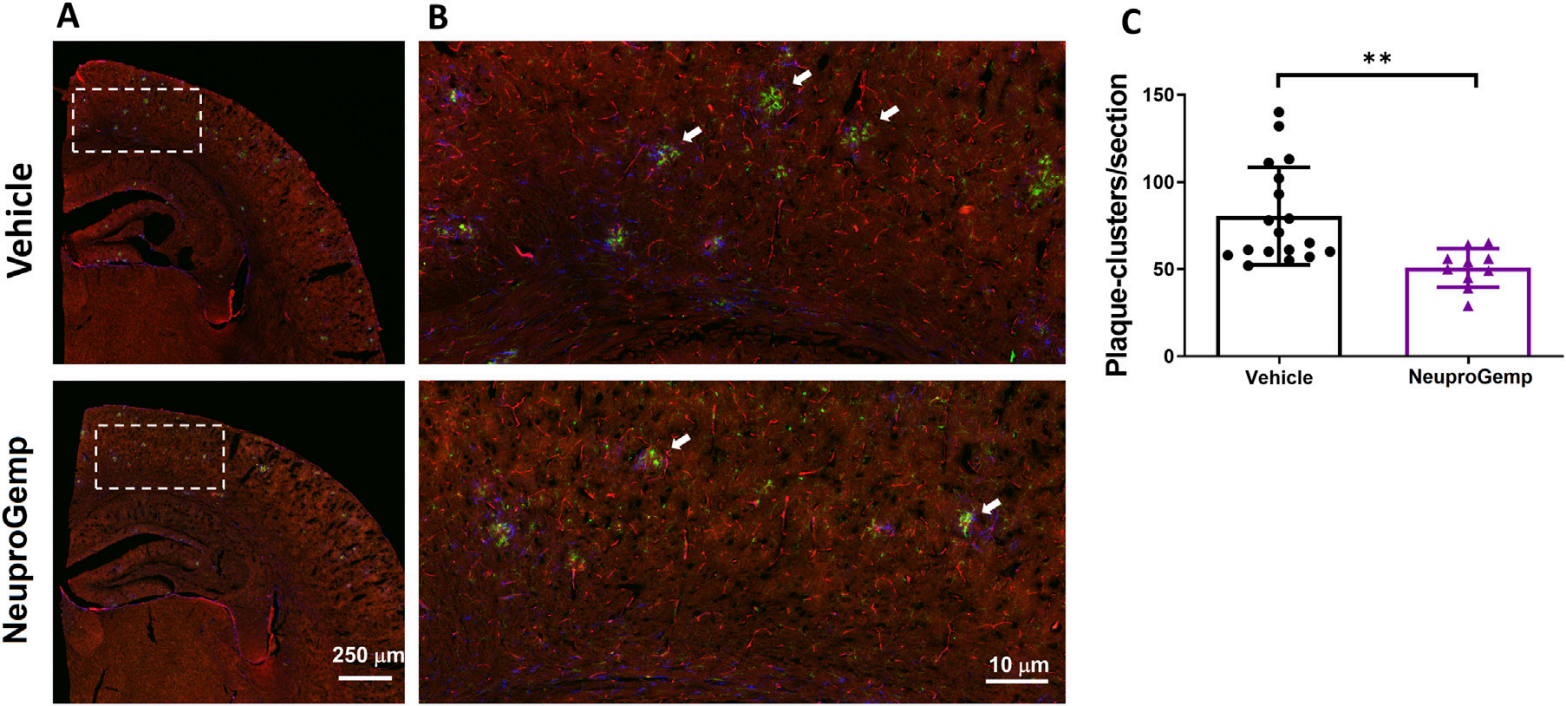
NeuproGemp reduces plaque-associated microglial and astroglial clustering in APP/PS1 mice. 5-month-old APP/PS1 transgenic mice were orally administered vehicle (n = 18), HE-A (30 mg/kg/day, n = 9), or NeuproGemp (300 mg/kg/day, n = 9) for 8 weeks. Amyloid plaques were visualized by immunohistochemical staining with AB-10 antibody (red). Microglia and reactive astrocytes were detected using Iba-1 antibody (green) and GFAP antibody (blue), respectively. **(A)** Representative immunofluorescence images of the parietal cortical area. Scale bar: 250 μm. **(B)** Magnified view of the boxed region in panel A, highlighting plaque-associated microglial and astroglial clusters (indicated by white arrows). Scale bar: 10 μm. **(C)** Quantification of microglial and astroglial clusters in cortical sections using image analysis software. Data are presented as mean ± SD. Statistical significance between Vehicle and NeuproGemp-treated groups was determined using unpaired two-tailed Student’s t-test, with **, p < 0.01. All sections were prepared and imaged under identical conditions, and investigators performing quantification were blinded to treatment groups.

To investigate the impact of NeuproGemp treatment on glial responses associated with amyloid plaques, we performed immunohistochemical analysis on brain sections from 5-month-old APP/PS1 transgenic mice treated with either vehicle or NeuproGemp (300 mg/kg/day) for 8 weeks. Amyloid plaques were labeled using the AB-10 antibody (red), microglia were identified by Iba-1 (green), and astrocytes by GFAP (blue). Low-magnification images of the parietal cortex (Figure 3A) revealed widespread amyloid deposition in both vehicle- and NeuproGemp-treated mice. However, higher magnification images of the boxed cortical regions (Figure 3B) showed clear differences in glial organization around plaques. In the vehicle-treated group, amyloid plaques were frequently surrounded by dense clusters of activated microglia and reactive astrocytes, as indicated by the co-localization of Iba-1 and GFAP immunoreactivity around plaques (white arrows). These glial clusters were less prevalent in the NeuproGemp-treated group, where the glial responses appeared more diffuse and less tightly associated with plaques. Quantitative analysis (Figure 3C) confirmed that the number of microglial and astroglial clusters associated with plaques was significantly reduced in the NeuproGemp group compared to vehicle controls. On average, vehicle-treated mice exhibited approximately 95 ± 22 clusters per section, while NeuproGemp-treated mice showed a significantly lower number, approximately 55 ± 11 clusters per section (p < 0.01, Student's t test). These data suggest that NeuproGemp treatment attenuates the recruitment or retention of glial cells around amyloid plaques in this Alzheimer’s disease model.

### 3.4 Effect of NeuproGemp on hippocampal neurogenesis in APP/PS1 mice

**FIGURE 4:**
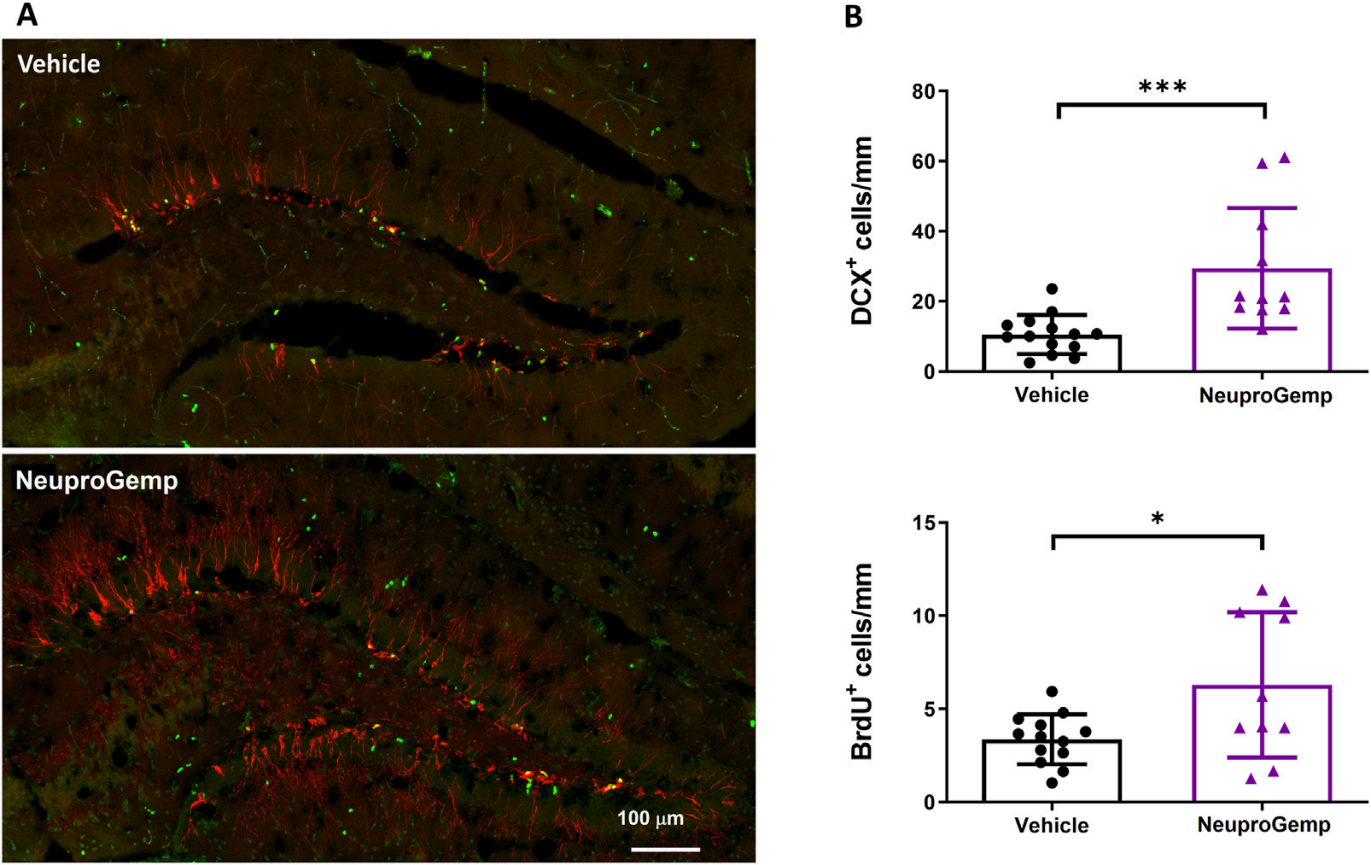
NeuproGemp enhances hippocampal neurogenesis in APP/PS1 mice. 5-month-old APP/PS1 transgenic mice were orally administered vehicle (n = 14) or NeuproGemp (300 mg/kg/day, n = 10) for 8 weeks. Hippocampal neurogenesis was assessed by immunofluorescence staining with doublecortin (DCX, red) for immature neurons and BrdU (green) for proliferating cells. **(A)** Representative images of the dentate gyrus (DG) area. Scale bar: 100 μm. **(B)** Quantification of DCX-positive and BrdU-positive cells in the subgranular zone (SGZ) of the DG, expressed as number of positive cells per mm of SGZ. Data are presented as mean ± SD. Statistical significance between Vehicle and NeuproGemp-treated groups was determined using unpaired two-tailed Student’s t-test, with *, p < 0.05; ***, p < 0.001. All staining and imaging were performed under identical conditions, and investigators performing quantification were blinded to treatment groups.

To investigate the effect of NeuproGemp on hippocampal neurogenesis in APP/PS1 transgenic mice, we conducted immunohistochemical analyses of doublecortin (DCX), a marker of immature neurons, and 5-bromo-2-deoxyuridine (BrdU), a marker of proliferating cells, in the subgranular zone (SGZ) of the dentate gyrus after 7 weeks of treatment. 5-month-old APP/PS1 mice were orally administered with either vehicle or NeuproGemp (300 mg/kg/day) daily for 7 weeks. Representative fluorescence images of the dentate gyrus (Figure 4A) show that NeuproGemp treatment markedly increased the number of DCX-positive cells (red) and BrdU-positive cells (green) in the SGZ region compared to vehicle-treated controls. Quantitative analysis (Figure 4B) revealed a statistically significant increase in the number of DCX + cells in NeuproGemp-treated mice (mean: ∼49 cells/mm) relative to the vehicle group (mean: ∼18 cells/mm), with a p-value <0.001 (**). Likewise, the density of BrdU + cells was significantly elevated in the NeuproGemp group (mean: ∼8 cells/mm) compared to the vehicle group (mean: ∼4.5 cells/mm), reaching statistical significance with p < 0.05. These results indicate that chronic oral administration of NeuproGemp effectively enhances both neural progenitor proliferation and early neuronal differentiation within the hippocampus of APP/PS1 mice.

### 3.5 The anti-Aβ_1-42_ and anti-pE-Aβ_3-42_ formation activity of NeuproGemp

**FIGURE 5:**
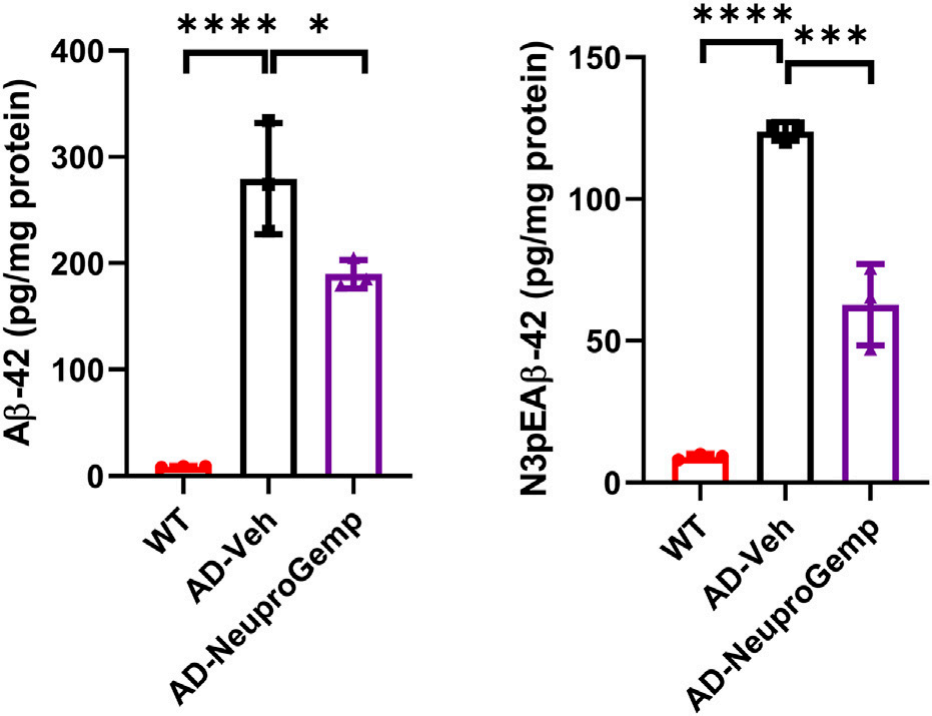
Effect of NeuproGemp on Aβ_1_–_42_ and pyroglutamate-Aβ_3_–_42_ (pE-Aβ_3_–_42_) levels in APP/PS1 mice. 5-month-old APP/PS1 transgenic mice were orally administered with vehicle (n = 3) or NeuproGemp (300 mg/kg/day, n = 3) for 8 weeks. Cortical and hippocampal tissues were harvested and homogenized, and the levels of Aβ_1_–_42_ and pE-Aβ_3_–_42_ were quantified using commercially available ELISA kits (Invitrogen, KHB3442; IBL, 27716) following the manufacturer’s protocols. Data are presented as mean ± SD. Statistical comparisons between Vehicle and NeuproGemp groups were performed using unpaired Student’s t-test, with significant differences indicated by *p < 0.05; **p < 0.01.

We further estimated the anti-amyloid beta (Aβ) formation activity of NeuproGemp. Specifically, APP/PS1 mice were administered a diet supplemented with NeuproGemp (300 mg/kg/day) for 8 weeks. Following this intervention, levels of soluble Aβ_1-42_ and pE-Aβ_3-42_ in the cerebral cortex of APP/PS1 mice were quantified using enzyme-linked immunosorbent assay (ELISA). Notably, NeuproGemp demonstrated a marked reduction in Aβ formation within the brains of treated mice. Quantitative analysis revealed that administration of NeuproGemp resulted in a reduction of approximately 30% in soluble Aβ_1-42_ levels compared to the control group receiving vehicle treatment (Figure 5). Additionally, the pE-Aβ_3-42_ levels were significantly decreased by 50% in the brains of AD mice treated with NeuproGemp when compared to those that received the vehicle control (Figure 5). These findings indicate that NeuproGemp effectively inhibits human glutaminyl cyclase (hQC) activity, leading to a substantial decrease in the production of pE-Aβ_3-42_. This suggests that NeuproGemp has the potential to attenuate or prevent the progression of Alzheimer’s disease pathology, thereby contributing to strategies aimed at mitigating neurodegeneration associated with this condition.

### 3.6 hQC inhibitory activity

**FIGURE 6:**
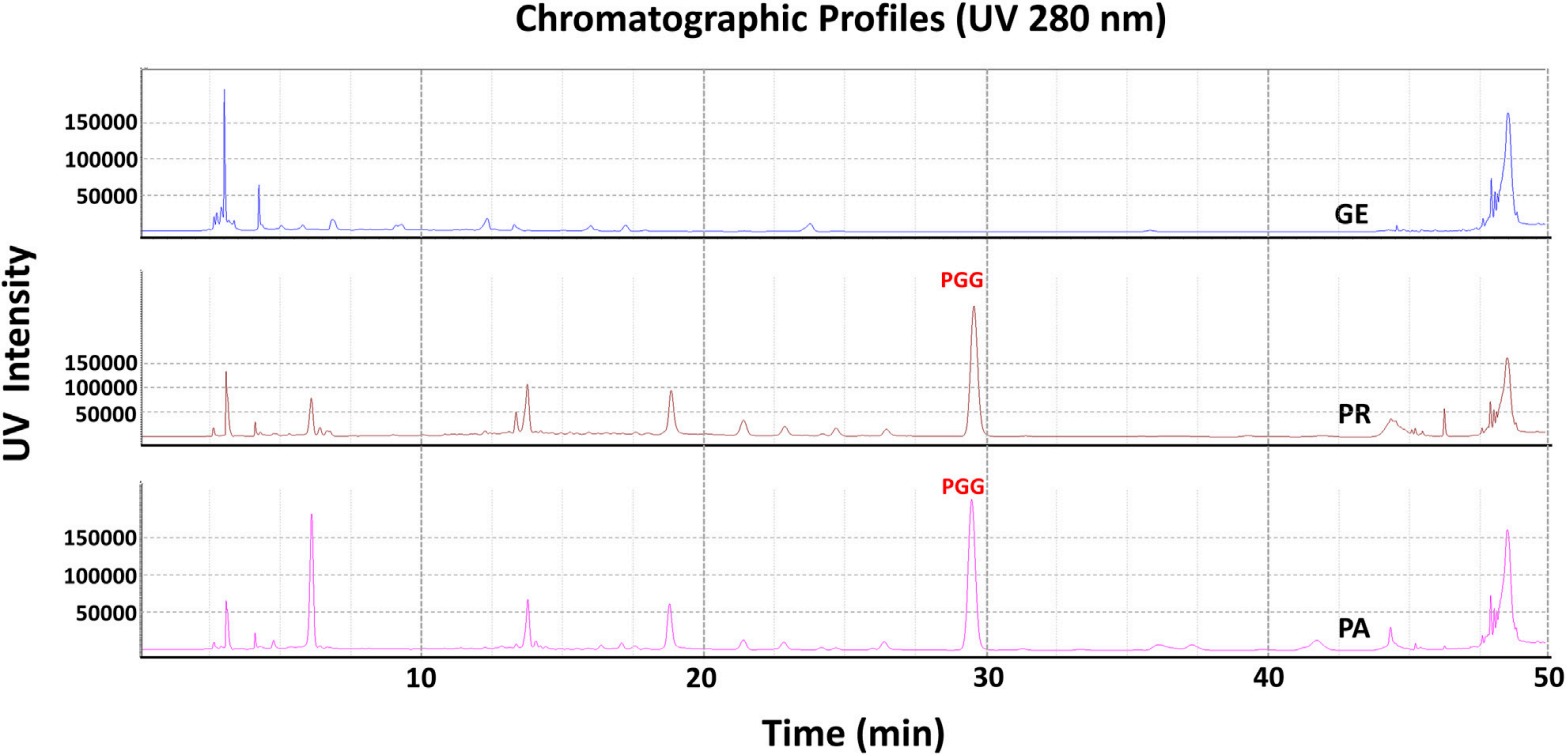
HPLC profile of pentagalloylglucose (PGG) in NeuproGemp and its constituent TCM herbs. Samples were extracted with 50% methanol (20 mL) by ultrasonication for 30 min, filtered, and diluted for analysis. Reference PGG (Sigma-Aldrich, 1 mg/mL stock in 50% methanol) was used for calibration. HPLC analysis was performed on a Shimadzu Nexera-i LC-2060C equipped with an ODS COSMOSIL 5C18-AR-II column (4.6 × 250 mm) at 35 °C. The mobile phase for PGG consisted of water with 0.1% phosphoric acid and acetonitrile with 0.1% phosphoric acid under a gradient: 0.01–7 min, 5%–10% B; 7–10 min, 10%–15% B; 10–22 min, 15%–18% B; 22–40 min, 18%–20% B; 40–45 min, 20%–100% B; 45–55 min, 100%. Flow rate was 1.0 mL/min, injection volume 1–10 μL, detection wavelength 280 nm. Peaks were identified by comparison with the PGG standard and quantified using calibration curves.

**FIGURE 7:**
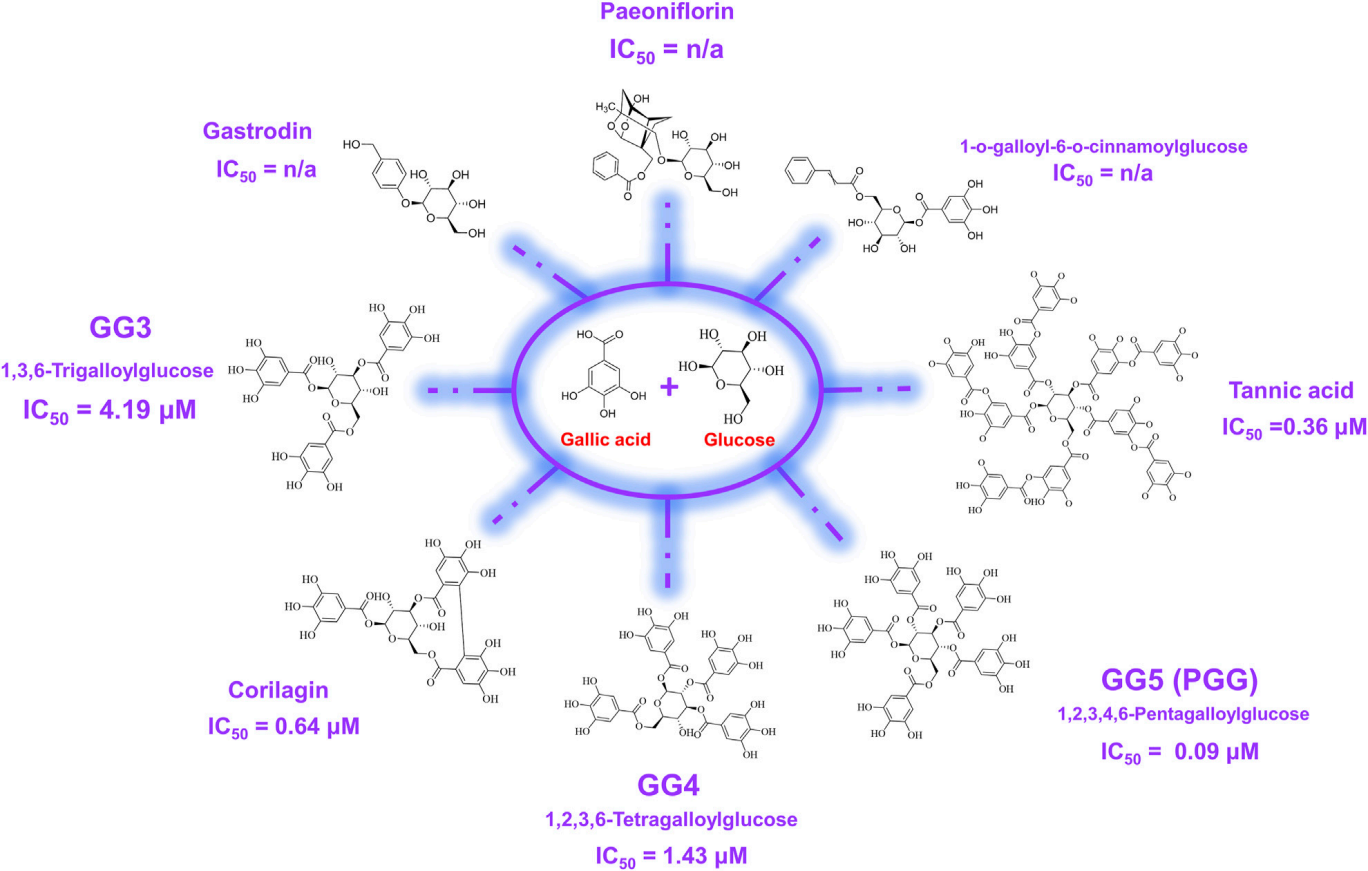
Chemical structures of pentagalloylglucose (PGG) and its galloyl derivatives. PGG consists of a β-D-glucose core esterified with five gallic acid (3,4,5-trihydroxybenzoic acid) moieties. Additional derivatives, including Corilagin, 1,3,6-trigalloylglucose (GG3), 1,2,3,6-tetragalloylglucose (GG4), and tannic acid, differ in the number and position of galloyl units attached to the glucose scaffold. These structural variations were evaluated in this study to assess their impact on human glutaminyl cyclase (hQC) inhibition and anti-pyroglutamate-Aβ activity.

**FIGURE 8:**
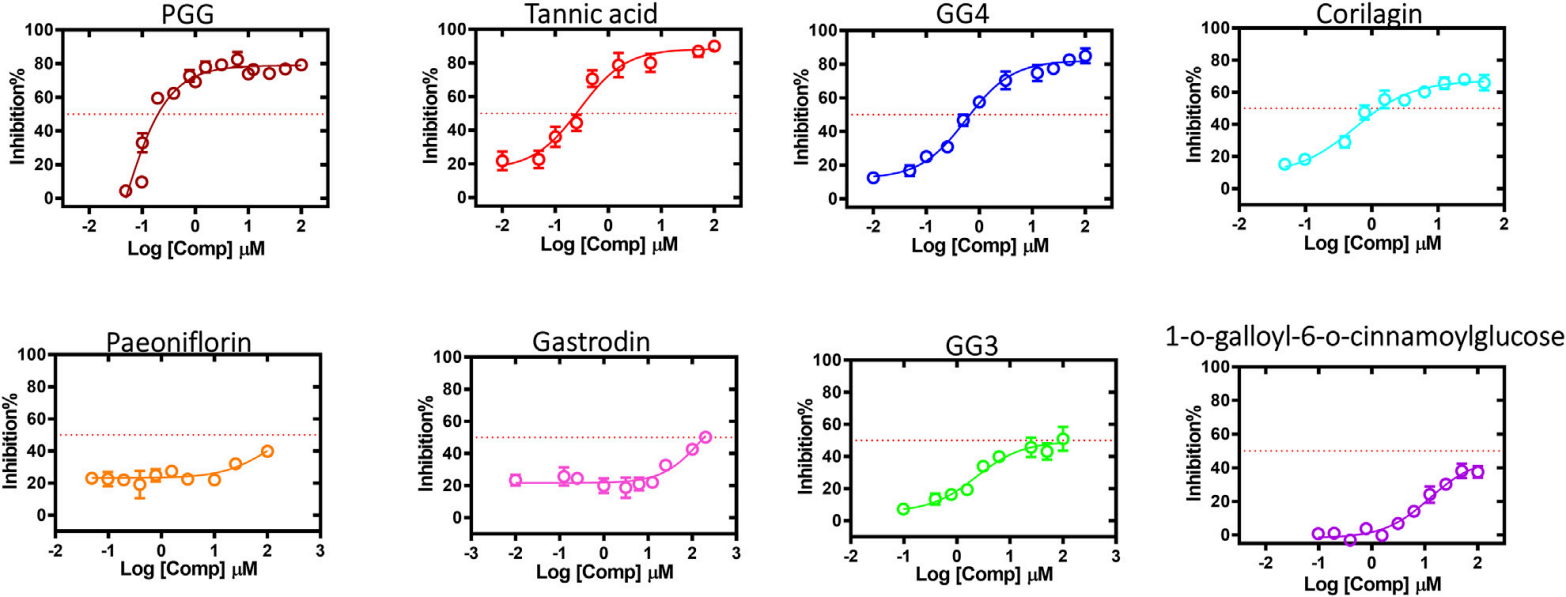
Inhibitory effects of pentagalloylglucose (PGG) and its derivatives on human glutaminyl cyclase (hQC) activity. The enzymatic activity of recombinant hQC was measured using a coupled spectrophotometric assay with H-Gln-Gln-OH as substrate and glutamic dehydrogenase as a coupling enzyme. PGG and its derivatives—including Corilagin, 1,3,6-trigalloylglucose (GG3), 1,2,3,6-tetragalloylglucose (GG4), and tannic acid—were tested at various concentrations to determine their inhibitory potency. Initial reaction rates were monitored by measuring NADH oxidation at 340 nm, and IC_50_ values were calculated using nonlinear regression in GraphPad Prism 6. Data are presented as mean ± SD from three independent experiments. Lower IC_50_ values indicate stronger inhibition of hQC activity.

In order to elucidate which component in NeuproGemp was chiefly responsible for the inhibition of hQC activity, we performed HPLC analysis and preliminary inhibition assays, through which we identified pentagalloylglucose (PGG) (Figure 6; Table 1), extracted from *Paeoniae Radix Rubra,* was the main bioactive component inhibiting the activity of hQC. To further investigate the inhibitory potency of PGG and its derivatives, we conducted comprehensive dose-response assays across a range of concentrations. The results demonstrated that PGG, tannic acid, and corilagin all exhibited strong, dose-dependent inhibition against hQC, with IC_50_ values of 0.09 ± 1.2, 0.36 ± 1.2, and 0.64 ± 1.5 µM, respectively (Figures 7, 8). These data suggested that PGG and its derivatives are potent inhibitors of hQC, highlighting their potential therapeutic relevance. In contrast, when we assessed the inhibitory activity of other derivatives—such as paeoniflorin, gastrodin, GG3 (1,3,6-Trigalloylglucose), and 1-o-galloyl-6-o-cinnamoylglucose—the results were markedly different (Figure 8). Gastrodin and GG3 showed only weak inhibitory effects on hQC, while paeoniflorin and 1-o-galloyl-6-o-cinnamoylglucose exhibited minimal to no significant inhibition. These findings suggest that the complete structure of PGG, or its key derivative forms like tannic acid and corilagin, is critical for the potent inhibition of hQC activity, while the individual subunits lack the necessary features to exert strong inhibitory effects on their own.

**TABLE 1:** Quantification of Pentagalloylglucose and related compounds using HPLC analysis.

The reference compound	Pentagalloylglucose
Retention Time	29.61 min
Regression Equation^a^	y = 31140x + 55102
R^2^ ^b^	0.9994
PA	2.931 ± 0.004 mg/g
PR	3.838 ± 0.005 mg/g
GE	N.D.
TC	1.238 ± 0.006 mg/g

^a^ in the regression equation: y = ax + b, x and y indicate the concentration (μg/mL) and peak area (mAU.s), respectively.

^b^ *R*2 is the correlation coefficient of the equation. N.D.: no detection.

### 3.7 The binding affinities of PGG and tannic acid towards hQC

**FIGURE 9:**
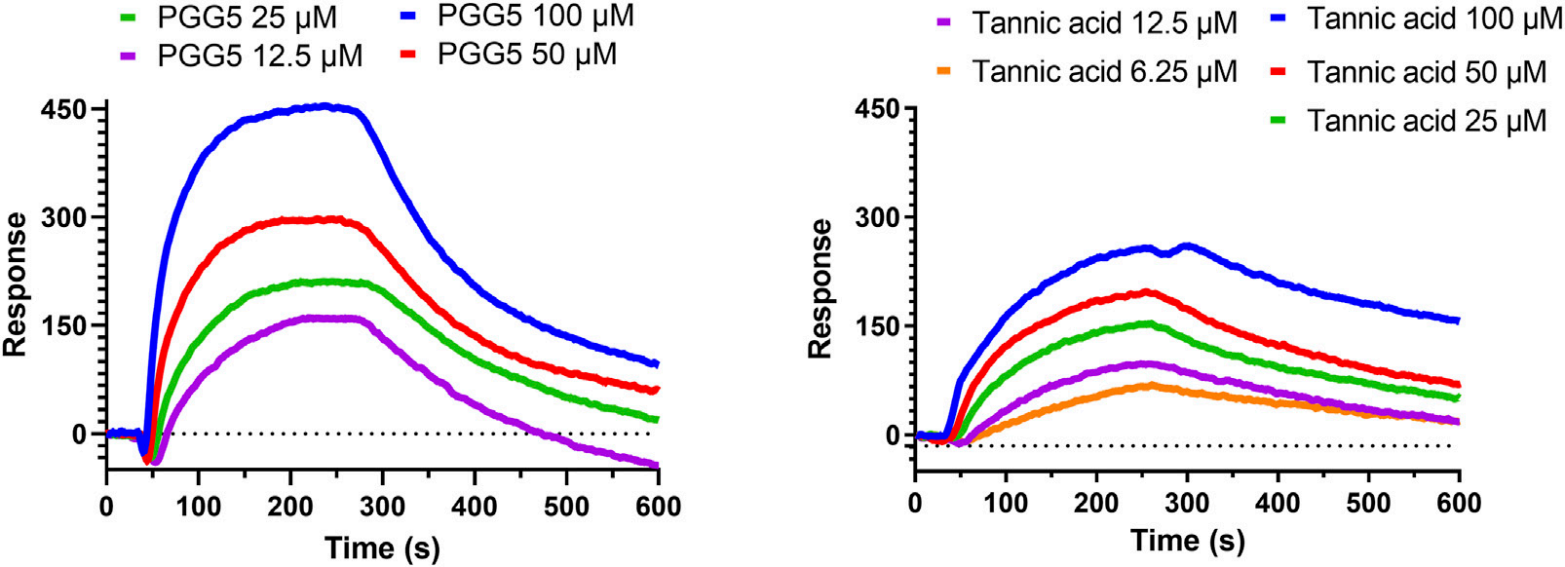
Binding of pentagalloylglucose (PGG) and tannic acid to human glutaminyl cyclase (hQC) assessed by localized surface plasmon resonance (LSPR). Recombinant hQC was immobilized on an NTA sensor chip, and varying concentrations of PGG or tannic acid were flowed over the chip in Tris-T buffer (50 mM Tris-HCl, pH 7.4, 150 mM NaCl, 0.005% Tween 20) containing 0.5% DMSO and 2% BSA. Association and dissociation were monitored in real time, and sensorgrams were fitted using a 1:1 binding model to calculate dissociation constants (K_D). Representative sensorgrams for PGG and tannic acid are shown. Data demonstrate high-affinity binding of both compounds to hQC, with K_D values indicating potent interactions. Experiments were performed in triplicate.

From the extract of *Paeoniae Radix Rubra*, we successfully identified PGG as a potent inhibitor of hQC, with its derivatives, tannic acid, exhibiting comparable inhibitory efficacy. To further substantiate the binding between these inhibitors and hQC, localized surface plasmon resonance (LSPR) experiments were performed. PGG was tested at concentrations of 12.5, 25, 50, and 100 μM during the LSPR analysis (Figure 9). The sensorgrams revealed a binding profile characterized by a slow association phase followed by rapid dissociation, indicative of a transient interaction between PGG and hQC. This interaction yielded a dissociation constant (KD) of 63.7 ± 0.1 nM (Figure 9). Similarly, the binding of tannic acid to hQC was evaluated at concentrations of 6.25, 12.5, 25, 50, and 100 μM (Figure 9). Although the binding signals for tannic acid were slightly weaker compared to PGG, the sensorgrams displayed a similar kinetic profile, with slow association and rapid dissociation, suggesting analogous binding behavior between tannic acid and hQC. Consequently, the KD value of tannic acid to hQC was determined to be 22.8 ± 0.1 nM.

### 3.8 Molecular modeling and dynamics simulations reveal the mode of actions of inhibitors against hQC

**FIGURE 10:**
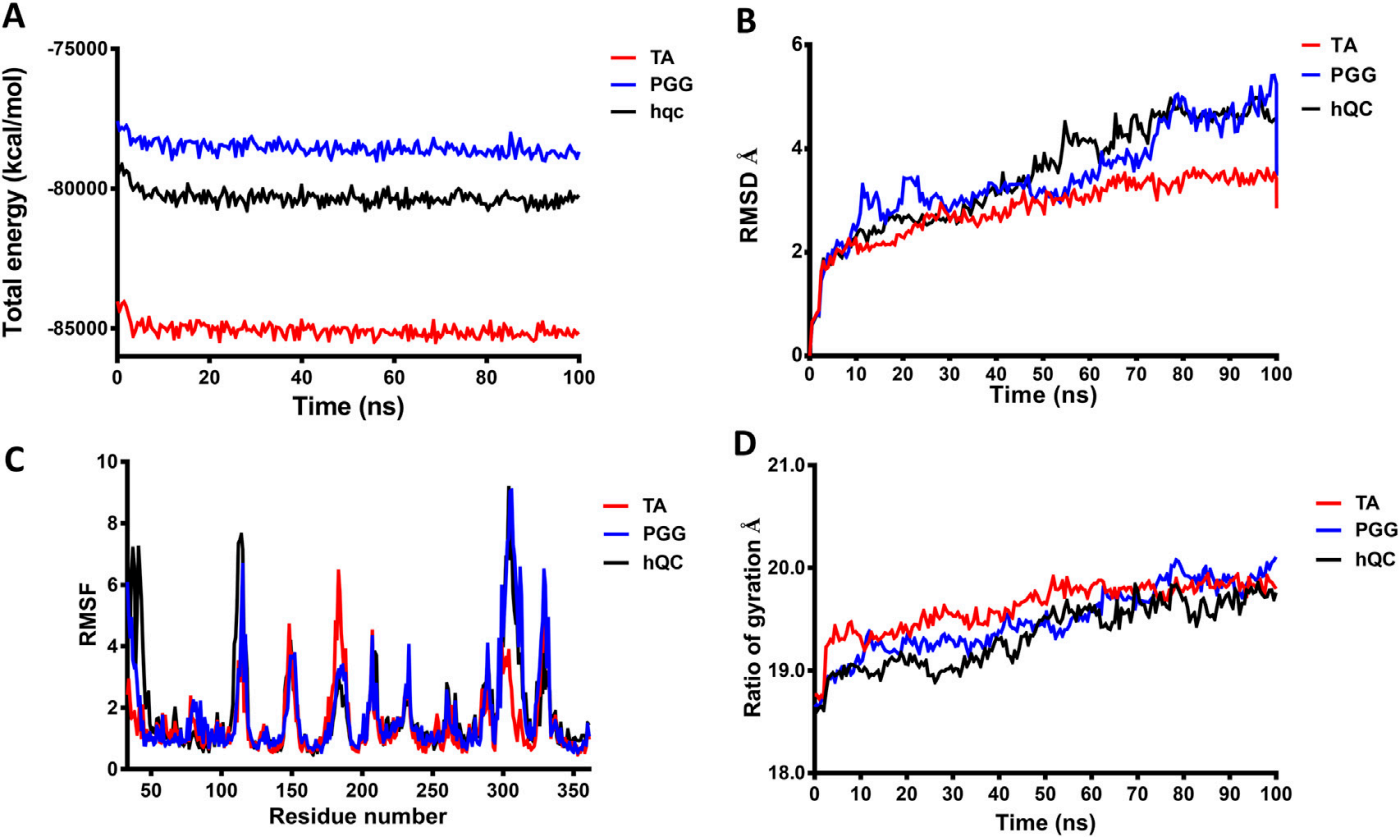
Molecular dynamics (MD) simulation analysis of hQC in complex with PGG and tannic acid. MD simulations of human glutaminyl cyclase (hQC) alone (apo) and in complex with PGG or tannic acid were performed for 100 ns using the CHARMM force field. **(A)** Total potential energy of the systems over the simulation time, indicating overall system stability. **(B)** Root-mean-square deviation (RMSD) of Cα atoms for apo-hQC and inhibitor-bound complexes, showing the conformational stability of the protein during the simulation. **(C)** Root-mean-square fluctuation (RMSF) of individual hQC residues, highlighting residue-specific flexibility and dynamic regions upon inhibitor binding. **(D)** Radius of gyration (Rg) values over time, reflecting the compactness and structural stability of the protein complexes. Data were derived from trajectory analyses of the last 100 ns of simulation.

To achieve a more comprehensive understanding of the atomic-level interactions between hQC and the identified inhibitors, molecular modeling approaches were employed to construct the protein-ligand complex structures. Key residues (H140, K144, F154, D159, E201, E202, W207, D248, L249, V302, I303, Q304, D305, I321, F325, P326, E327, W329, and H330) within the active site of hQC were utilized to define the binding site for subsequent docking studies. The binding interactions of PGG and tannic acid with hQC were examined using GOLD molecular docking software, with the aim of elucidating the underlying molecular mechanisms of inhibition. Eventually, we selected the model with the lowest energy as the final complex structure. The built complex structure was further subjected for 100 ns molecular dynamics (MD) simulations. MD simulations were performed to determine binding behaviors of the inhibitor to hQC. The average RMSD, RMSF, and Rg of hQC-PGG and hQC-tannic acid for all conformations were calculated through simulations with a duration of 100 ns and compared with those of the control (hQC alone) to determine the stability of the complexes. The total energies of hQC alone and in complex with inhibitors as a function of simulation times were shown in Figure 10A. The average RMSD for hQC alone was 3.52 Å, higher than that of the hQC-PGG complex (3.48 Å) and hQC-tannic acid (2.85 Å) (Figure 10B). Fluctuations in individual residues within the simulation period are plotted in Figure 10C (RMSF). The residues for hQC, hQC-PGG, and hQC-tannic acid had similar RMSF patterns, suggesting that these ligands did not considerably affected the stability of hQC and altered its dynamic behavior. The Rg for hQC was more stable, and fluctuated slightly between 18.6 and 19.9 Å (Figure 10D). Similarly, for hQC-tannic acid, Rg fluctuated between 18.7 and 19.9 Å. By contrast, Rg values of hQC-PGG slightly fluctuated between 18.6 and 20.1 Å. These results implied that tannic acid formed a more thermodynamically stable complex with hQC than PGG.

### 3.9 Analyses of molecular interactions of inhibitors targeting hQC

**FIGURE 11:**
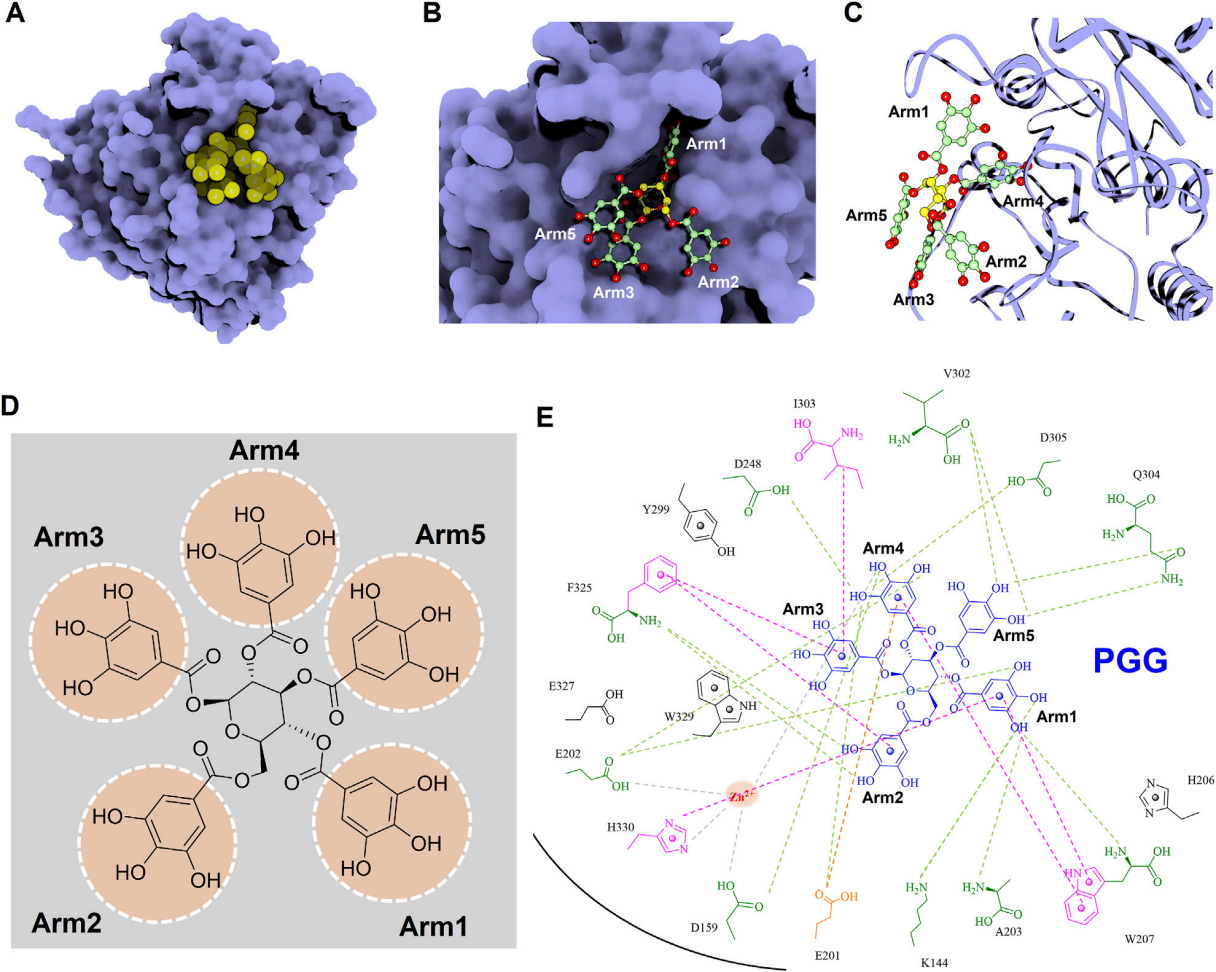
Detailed molecular interactions between PGG and human glutaminyl cyclase (hQC) revealed by molecular docking and MD simulations. **(A)** Surface representation of hQC (purple) with PGG shown as yellow spheres, illustrating the overall binding site. **(B)** Magnified view of the hQC–PGG interface with PGG in ball-and-stick representation and hQC in surface mode, highlighting key contact regions. **(C)** PGG displayed as ball-and-stick and hQC as a ribbon structure (purple) to show the spatial orientation of the binding pocket and ligand accommodation. **(D)** Chemical structure of PGG, depicting its pentagalloylglucose scaffold. **(E)** 2D interaction diagram between PGG and hQC, where green dashed lines indicate hydrogen bonds, magenta dashed lines represent hydrophobic contacts, orange dashed lines show anion–π interactions, and gray dashed lines denote coordination with the catalytic zinc ion. These analyses identify critical residues involved in PGG binding and provide insight into its inhibitory mechanism against hQC.

**FIGURE 12:**
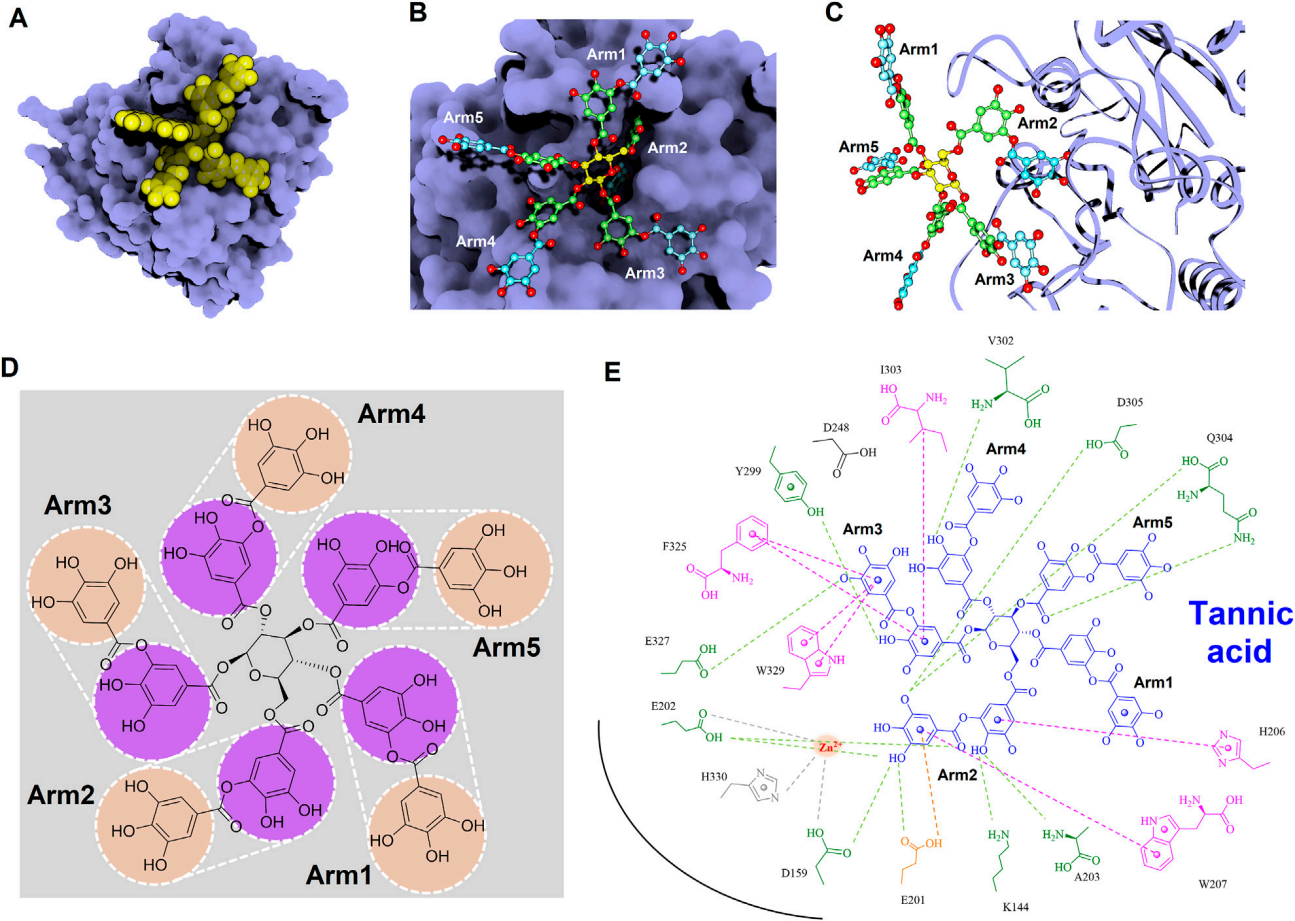
Detailed molecular interactions between tannic acid and human glutaminyl cyclase (hQC) revealed by molecular docking and MD simulations. **(A)** Surface representation of hQC (purple) with tannic acid shown as yellow spheres, illustrating the overall binding site. **(B)** Magnified view of the hQC–tannic acid interface with tannic acid in ball-and-stick representation and hQC in surface mode, highlighting key contact regions. **(C)** Tannic acid displayed as ball-and-stick and hQC as a ribbon structure (purple) to show the spatial orientation of the binding pocket and ligand accommodation. **(D)** Chemical structure of tannic acid, depicting its polyphenolic scaffold. **(E)** 2D interaction diagram between tannic acid and hQC, where green dashed lines indicate hydrogen bonds, magenta dashed lines represent hydrophobic contacts, orange dashed lines show anion–π interactions, and gray dashed lines denote coordination with the catalytic zinc ion. These analyses identify critical residues involved in tannic acid binding and provide insight into its inhibitory mechanism against hQC.

To gain deeper insights into the molecular interactions between the identified inhibitors—PGG and tannic acid—and human glutaminyl cyclase (hQC), structural analyses were conducted using the non-bond interaction analysis module in Discovery Studio 2021. Representative binding conformations of both ligands in complex with hQC were extracted from the molecular dynamics (MD) simulation at the 100 ns time point. The results revealed that PGG and tannic acid occupied distinct positions and orientations within the active site of hQC, establishing interactions with catalytically relevant residues (Figures 11, 12). Both ligands formed a range of non-covalent interactions, including electrostatic attractions, hydrogen bonds, and hydrophobic contacts. Specifically, PGG engaged in 15 hydrogen bonds, 6 hydrophobic interactions, one Zn^2+^ coordination, and one anion–π interaction with residues in the hQC active site. In comparison, tannic acid formed 12 hydrogen bonds, 7 hydrophobic contacts, one Zn^2+^ coordination, and one anion–π interaction at the same simulation time point. These findings suggest that both compounds exhibit stable and multifaceted binding to hQC, potentially contributing to their inhibitory activities.

## 4 Discussion

Alzheimer’s disease (AD) is a progressive neurodegenerative disorder and the most prevalent cause of dementia worldwide, currently affecting over 40 million individuals and anticipated to double in incidence over the next 2 decades (Fan et al., 2019; Livingston et al., 2020). The neuropathological hallmarks of AD include extracellular amyloid-beta (Aβ) deposition and the intracellular accumulation of hyperphosphorylated tau, which together drive synaptic dysfunction and neuronal loss (Murphy and LeVine, 2010; Mondragon-Rodriguez et al., 2012; Chen et al., 2017). Several TCM-derived herbs have demonstrated modulatory effects on oxidative stress, neuroinflammation, neurotransmitter signaling, and synaptic plasticity (Liu et al., 2020; Moreira et al., 2023; Chen et al., 2024; Jin et al., 2024; Meng et al., 2025). Despite these findings, the direct interactions of such herbal compounds with specific AD-associated pathomechanisms—particularly Aβ_1-42_ aggregation and hQC-mediated pE-Aβ_3-42_ formation—remain poorly characterized. Importantly, while targeting Aβ deposition may slow disease progression, it is unlikely to reverse pre-existing neuronal degeneration. As such, the development of therapeutic agents that concurrently inhibit Aβ pathology and activate endogenous neurorestorative mechanisms (e.g., neurogenesis, synaptogenesis, and axonal repair) represents a critical unmet need. TCM-derived compounds, with their inherent polypharmacological properties, are uniquely positioned to address this therapeutic gap. Systematic investigation into how these compounds modulate Aβ species formation, hQC activity, and regenerative signaling pathways is essential for advancing them toward clinical translation. In this study, we demonstrated that NeuproGemp treatment in 5–6-month-old APP/PS1 mice significantly reduced Aβ deposition and plaque-associated gliosis, improved hippocampal neurogenesis, and enhanced behavioral performance. These findings are consistent with the role of human glutaminyl cyclase (hQC) in catalyzing the formation of highly neurotoxic pE-Aβ species, as inhibition of hQC by PGG leads to decreased pE-Aβ accumulation, aligning with prior studies on hQC inhibitors in APP/PS1 mice (Chen D. et al., 2023). Although tau pathology was not directly assessed in the present study, early Aβ deposition in APP/PS1 mice has been shown to trigger tau hyperphosphorylation and neurofibrillary tangle formation, suggesting that interventions reducing Aβ burden may indirectly mitigate tau-associated pathology (Sanchez-Varo et al., 2021). By attenuating plaque-associated microglial and astroglial clustering, NeuproGemp also modulated neuroinflammatory responses, consistent with the anti-inflammatory and antioxidant properties of polyphenols, including PGG and tannic acid (Kabir et al., 2023). Taken together, these results suggest that NeuproGemp exerts multi-faceted neuroprotective effects in APP/PS1 mice by simultaneously reducing amyloidogenic stress, mitigating neuroinflammatory responses, and potentially influencing downstream tau pathology. This highlights the potential of NeuproGemp as a multi-targeted intervention for early-stage AD.

We provide comprehensive preclinical evidence supporting the therapeutic potential of the traditional Chinese medicine (TCM)-derived formulation NeuproGemp in attenuating Alzheimer’s disease (AD)-like phenotypes in the APP/PS1 transgenic mouse model. Behavioral assessments demonstrated that chronic oral administration of NeuproGemp over 8 weeks significantly ameliorated hippocampal-dependent spatial learning and memory impairments, as evidenced by enhanced performance in the Morris water maze (MWM) task (Figure 2). NeuproGemp-treated mice exhibited reduced escape latency during the acquisition phase and demonstrated a higher frequency of direct navigation to the hidden platform in the probe trial (Figure 2), indicative of improved spatial memory acquisition and retrieval—functions critically mediated by hippocampal circuits. In addition to cognitive enhancement, NeuproGemp significantly restored burrowing activity (Figure 1), a robust ethologically relevant behavior reflective of general wellbeing and motivational status. Given that disruptions in burrowing are often associated with neurodegenerative processes affecting limbic, motor, and motivational systems, this improvement suggests that NeuproGemp may exert broader neurobehavioral benefits beyond cognition, potentially through modulation of affective and motivational neural pathways. In contrast, performance in the novel object recognition (NOR) test showed only a non-significant trend toward improvement, potentially reflecting a region-specific effect of NeuproGemp favoring hippocampal over perirhinal cortex function, or limitations inherent to the NOR paradigm under the current experimental conditions. Histopathological analysis further revealed that NeuproGemp markedly reduced glial clustering—primarily reactive microglia and astrocytes—around amyloid plaques in the parietal cortex (Figure 3). This reduction in plaque-associated gliosis, a hallmark of chronic neuroinflammation in AD, may indicate a shift toward a less neurotoxic glial phenotype, potentially through attenuation of innate immune signaling or a reduction in the immunogenicity of amyloid aggregates. Significantly, NeuproGemp apparently promotes the proliferation of neural progenitor cells and facilitates early-stage neuronal differentiation in the hippocampus of APP/PS1 transgenic mice (Figure 4). Our findings that PGG directly inhibits hQC and reduces pE-Aβ formation are in agreement with recent reports underscoring hQC as a validated therapeutic target for attenuating the generation of highly neurotoxic Aβ species in AD (Chen D. et al., 2023). Notably, the choice of 5–6-month-old APP/PS1 mice is supported by previous studies demonstrating that early synaptic alterations, neuroinflammatory activation, and measurable cognitive deficits emerge as early as 4.5 months of age (Sanchez-Varo et al., 2021), thereby providing a critical window to evaluate prophylactic interventions. Furthermore, our results converge with recent comprehensive reviews that emphasize the multi-targeted actions of dietary polyphenols—including anti-amyloid, anti-inflammatory, antioxidant, and synaptoprotective effects—in mitigating AD-related pathology (Kabir et al., 2023). Importantly, our study extends these insights by identifying PGG as a key bioactive constituent of NeuproGemp that mechanistically links polyphenol activity with hQC inhibition, offering a novel avenue for multi-component botanical interventions against AD.

In a complementary biochemical investigation, we further evaluated the effect of NeuproGemp on pyroglutamate-modified amyloid-β (pE-Aβ_3-42_), a particularly neurotoxic species implicated in AD pathogenesis. NeuproGemp is a multi-component herbal mixture consisting of *G. elata*, *Paeoniae Radix Rubra*, and GMI (Ganoderma microsporum immunomodulatory protein). Quantification of Aβ species by immunoassays revealed that NeuproGemp administration significantly reduced both Aβ_1-42_ and pE-Aβ_3-42_ levels in the cortex and hippocampus of APP/PS1 mice (Figure 5). Among its constituents, GMI has been reported to exert neuroprotective effects by attenuating oxidative stress, modulating neuroinflammatory cytokine release, and preventing apoptosis. However, its limited blood–brain barrier (BBB) permeability poses challenges for direct central nervous system (CNS) efficacy. To identify small-molecule BBB-permeable bioactive components responsible for the observed effects, high-performance liquid chromatography (HPLC) (Figure 6) was employed to fractionate NeuproGemp, leading to the identification of pentagalloylglucose (PGG)—a polyphenolic compound derived from *Paeoniae Radix Rubra*. Enzymatic assays confirmed that PGG potently inhibits hQC activity (Figures 7, 8), suggesting a mechanistic link to the suppression of pE-Aβ_3-42_ production. Accordingly, these findings demonstrate that NeuproGemp exerts multi-faceted therapeutic effects in an AD mouse model, encompassing cognitive enhancement, restoration of ethologically relevant behaviors, and modulation of neuroinflammation and Aβ-related pathology. The identification of PGG as an hQC inhibitor further supports the rationale for developing multitargeted TCM-based interventions for AD. Future studies integrating transcriptomic profiling, cytokine quantification, and BBB-permeability assessments will be essential to fully elucidate the molecular mechanisms underlying NeuproGemp’s neuroprotective properties and to advance its translational potential.

The structural features of 1,2,3,4,6-penta-O-galloyl-β-D-glucose (PGG), characterized by a glucose core esterified with five galloyl groups (3,4,5-trihydroxybenzoic acid), provide a highly polyphenolic framework conducive to extensive molecular interactions (Figure 7). This dense arrangement of catechol-type moieties is hypothesized to favor strong binding with metal-dependent enzymes such as human glutaminyl cyclase (hQC), a key contributor to the generation of pyroglutamate-modified amyloid-β (pE-Aβ_3-42_), which plays a critical role in the pathogenesis of Alzheimer’s disease. To examine the structure–activity relationships among gallic acid derivatives, we evaluated a panel of natural compounds for their ability to inhibit hQC. Notably, Corilagin, 1,3,6-trigalloylglucose (GG3), 1,2,3,6-tetragalloylglucose (GG4), PGG, and tannic acid exhibited strong inhibitory effects, with IC_50_ values in the submicromolar to low micromolar range (0.64, 4.19, 1.43, 0.09, and 0.36 μM, respectively) (Figure 7). In contrast, compounds containing fewer phenolic groups or with bulkier, less polar substituents—such as Gastrodin, Paeoniflorin, and 1-O-galloyl-6-O-cinnamoylglucose—showed limited inhibition, with less than 50% activity suppression at 100 μM. These findings underscore the significance of both galloyl unit number and molecular compactness in promoting effective hQC inhibition.

To further elucidate the inhibitory mechanisms of the two most potent compounds, PGG and tannic acid, we conducted molecular docking followed by 100-ns molecular dynamics (MD) simulations. Root-mean-square deviation (RMSD) analysis indicated that binding of either compound slightly stabilized the hQC conformation relative to the apo form, with RMSD values of 3.48 Å (PGG–hQC) and 2.85 Å (tannic acid–hQC) compared to 3.52 Å for the unbound enzyme (Figure 10B). This suggests that both inhibitors contribute to maintaining global structural stability upon binding. Further analysis of root-mean-square fluctuation (RMSF) revealed that local residue flexibility was not substantially altered in either complex (Figure 10C), implying that the ligand binding did not disrupt native dynamic behavior of the enzyme. Consistent with these findings, the radius of gyration (Rg) values remained relatively stable across all simulations, ranging between 18.6 and 19.9 Å for apo-hQC and hQC–tannic acid. In the case of hQC–PGG, slightly higher Rg fluctuations (up to 20.1 Å) were observed (Figure 10D), which may reflect minor conformational breathing induced by the compact but rigid nature of the PGG scaffold. Together, these computational results suggest that both PGG and tannic acid are capable of stably engaging the hQC catalytic domain without inducing deleterious conformational rearrangements. However, the observed differences in Rg dynamics hint at possible differences in binding-induced protein flexibility that may contribute to the differential potency observed experimentally. Non-bonded interaction analyses elucidated the molecular determinants of binding. PGG exhibited strong coordination to the catalytic zinc ion via catechol groups and established a dense hydrogen bonding network with K144, D159, E202, A203, W207, D248, V302, Q304, D305, and F325 (Figure 11). It also engaged in hydrophobic contacts with residues such as A203, W207, I303, F325, and H330, and formed anion–π interactions with E201. Tannic acid similarly coordinated the zinc ion and formed hydrogen bonds with K144, D159, E201, E202, A203, Y299, V302, Q304, D305, and E327, in addition to hydrophobic interactions with H206, W207, I303, F325, and W329. Anion–π interactions with E201 were also observed (Figure 12). Although both compounds share comparable interaction networks, the additional galloyl units in tannic acid—five of which extend beyond the immediate binding interface—may contribute to increased molecular flexibility, which could account for its slightly reduced inhibitory potency relative to PGG.

Tannic acid, a hydrolyzable polyphenol abundantly present in grapes, chocolate, red wine, and coffee, has been widely recognized for its diverse pharmacological activities across multiple biomedical domains (Chung et al., 1998; King and Young, 1999). In veterinary applications, dietary inclusion of tannic acid at concentrations ranging from 0.2% to 1.0% has been reported to significantly alleviate post-weaning diarrhea in livestock, thereby supporting its role as a functional additive for promoting animal health and resilience (Yu et al., 2020; Song et al., 2021). Beyond its veterinary utility, tannic acid has demonstrated promising anticancer properties, exhibiting cytotoxicity against a spectrum of tumor types including hepatocellular carcinoma, breast cancer, and colorectal cancer (Athar et al., 1989; Horikawa et al., 1994; Wu et al., 2004). These anticancer effects are largely mediated through its robust antioxidant capacity, which facilitates redox regulation, epigenetic modulation, and inhibition of oncogenic signaling cascades, positioning tannic acid as a potential chemopreventive agent. Importantly, tannic acid has been shown to enhance the efficacy of doxorubicin, a widely used anthracycline chemotherapeutic, while simultaneously mitigating its cardiotoxicity—a limiting adverse effect—thus outperforming conventional antioxidants such as butylated hydroxyanisole (BHA) and butylated hydroxytoluene (BHT) in preclinical evaluations (Tikoo et al., 2011; Zhang et al., 2017; Zheng et al., 2023). These dual therapeutic and protective actions underscore the compound’s multifaceted pharmacological potential. In addition to its anticancer activity, tannic acid also exerts broad-spectrum antimicrobial effects. It inhibits the growth of methicillin-resistant *Staphylococcus aureus* (MRSA) through disruption of resistance-associated pathways (Akiyama et al., 2001; Dong et al., 2018; Kirmusaoglu, 2019), and exhibits antiviral activity against enveloped viruses, including influenza A and herpes simplex virus type 2 (HSV-2). Notably, synergistic enhancement of antiviral efficacy has been observed when tannic acid is used in combination with silver nanoparticles, indicating potential for combinatory therapeutic strategies (Orlowski et al., 2014; Orlowski et al., 2018). Within the context of Alzheimer’s disease, the present study identifies tannic acid as a potent inhibitor of human glutaminyl cyclase (hQC), an enzyme critically involved in the formation of neurotoxic pyroglutamate-modified amyloid-β (pE-Aβ_3-42_). Tannic acid exhibited significant inhibitory activity, with an IC_50_ value of 0.36 ± 1.2 μM (Figure 7) and a dissociation constant (KD) of 22.8 ± 0.1 nM (Figure 9), demonstrating high binding affinity and functional potency against hQC. These findings are corroborated by molecular docking and interaction profiling analyses, which revealed that tannic acid engages the hQC active site through an extensive network of hydrogen bonds, hydrophobic contacts, and electrostatic interactions, stabilizing the inhibitor–enzyme complex and likely obstructing substrate access or catalytic turnover (Figure 12).

Our findings also substantiate the multifaceted pharmacological profile of 1,2,3,4,6-penta-O-galloyl-β-D-glucose (PGG), highlighting its potential as a promising natural scaffold for therapeutic development (Abdelwahed et al., 2007; Lin et al., 2011). Structurally characterized by five gallic acid moieties esterified to a β-D-glucose core, PGG demonstrates a configuration conducive to robust biological activity. Notably, its antioxidant capacity, as measured by radical-scavenging efficiency, surpasses that of its major metabolite, gallic acid (IC_50_ = 7.1 ± 0.26 µM vs. 12.1 ± 0.16 µM, respectively) (Shaikh et al., 2016), suggesting a synergistic effect arising from multivalency and enhanced electron-donating potential. In addition to its antioxidative capacity, PGG exhibits broad-spectrum antimicrobial activity. Its efficacy against drug-resistant pathogens such as *S. aureus* (Jiamboonsri et al., 2011), as well as its inhibition of viral entry and gene expression in HSV-1 and IAV models (Haid et al., 2015; Jin et al., 2016), implies a mechanism involving membrane interaction or host-cell entry blockade, although the precise molecular targets remain to be elucidated. These effects underscore the relevance of PGG as a candidate for infectious disease management, particularly under the growing threat of antimicrobial resistance. The antineoplastic properties of PGG are also of considerable interest. Previous studies have demonstrated its ability to induce apoptosis, inhibit tumor growth, and suppress metastasis, with evidence pointing to the inhibition of fatty acid synthase as a central mechanism (Li et al., 2011; Zhao et al., 2014; Deiab et al., 2015). This aligns with the growing recognition of lipid metabolism as a vulnerability in cancer cells, positioning PGG as a potential metabolic disruptor with pleiotropic effects on tumor progression. The current study identifies PGG as a highly potent inhibitor of hQC, with an IC_50_ value of 0.09 ± 1.2 µM (Figure 7). This is a significant finding given the pivotal role of hQC in the post-translational modification of amyloidogenic peptides, including pyroglutamate-Aβ formation—a pathogenic hallmark in Alzheimer’s disease. Surface plasmon resonance analysis further confirms the high binding affinity of PGG for hQC (KD = 63.7 ± 0.1 nM, Figure 9), supporting a direct and specific interaction. Structural modeling reveals that this binding is stabilized through an intricate network of hydrogen bonding, hydrophobic contacts, and electrostatic interactions, collectively contributing to the high inhibitory potency observed. Moreover, the comparative analysis with tannic acid suggests that both compounds share a common binding mode characterized by multipoint attachment and potential zinc coordination within the hQC active site. This shared pharmacophore model may serve as a basis for rational design of novel hQC inhibitors with improved pharmacokinetic and pharmacodynamic profiles. Beyond neurodegeneration, the observed anti-inflammatory effects of PGG—via inhibition of neutrophil chemotaxis and L-selectin-mediated adhesion (Jang et al., 2013; Kiss et al., 2013; Zhao et al., 2015)—further extend its therapeutic relevance to diseases characterized by aberrant immune responses, such as atherosclerosis and inflammatory bowel disease. Similarly, in metabolic disease models, PGG enhances insulin receptor activation and glucose uptake (Ren et al., 2006; Bruno et al., 2013), supporting its utility in glycemic regulation and diabetes management.

While our study provides evidence that NeuproGemp exerts neuroprotective effects in APP/PS1 mice, several limitations should be acknowledged. First, tau pathology was not directly assessed; therefore, the influence of NeuproGemp on tau hyperphosphorylation and neurofibrillary tangle formation remains speculative. Second, the experiments were conducted in a single transgenic AD model, limiting generalizability across other AD mouse models or human disease contexts. Third, behavioral assessments were not performed blindly, which may introduce observer bias. Finally, although we observed hQC inhibition and reductions in pE-Aβ levels, direct measurements of hQC activity in brain homogenates were not performed; thus, the causal link between PGG-mediated hQC inhibition and observed cognitive benefits requires further validation. Potential mechanisms underlying the observed effects of NeuproGemp may involve multi-targeted actions. PGG and other polyphenols likely reduce amyloidogenic stress by inhibiting hQC, thereby lowering pE-Aβ formation. In addition, the anti-inflammatory and antioxidant properties of polyphenols may attenuate plaque-associated microglial and astroglial activation, preserving neuronal integrity and promoting hippocampal neurogenesis. Collectively, these multi-faceted effects may contribute to improved spatial learning, memory, and species-specific behaviors in APP/PS1 mice, supporting the rationale for early-stage intervention in AD. Collectively, our study identifies NeuproGemp as a scientifically validated traditional Chinese medicine formulation with significant therapeutic potential for Alzheimer’s disease (AD). By elucidating its mechanism of action, we demonstrate that NeuproGemp exerts its neuroprotective effects, at least in part, through the inhibition of glutaminyl cyclase (hQC)—a critical enzyme in the formation of neurotoxic pyroglutamate-modified Aβ (pE-Aβ_3-42_). Administration of NeuproGemp markedly reduced cortical and hippocampal levels of both Aβ_1-42_ and pE-Aβ_3-42_ in APP/PS1 mice, highlighting its efficacy in attenuating amyloidogenic pathology *in vivo*. Through a combination of biochemical and computational analyses, we identified PGG as the primary active compound responsible for hQC inhibition, with tannic acid also displaying potent activity. Beyond their enzyme-inhibitory effects, both PGG and tannic acid possess a broad spectrum of biological activities—including antioxidative, anti-inflammatory, and metabolic modulation—that collectively contribute to multitargeted neuroprotective profile of NeuproGemp. These findings not only bridge traditional Chinese medicine with contemporary molecular pharmacology but also underscore the promise of NeuproGemp as a holistic and mechanism-based intervention for AD. Future translational research focused on clinical development, dosage formulation, and long-term safety will be critical to advancing NeuproGemp and its active constituents toward therapeutic application in human neurodegenerative diseases.

## 5 Conclusion

Our study tested the hypothesis that NeuproGemp, a traditional Chinese medicine formulation containing *G. elata*, *Paeoniae Radix Rubra*, and GMI, could mitigate Alzheimer’s-like pathology in APP/PS1 mice. Our findings demonstrate that chronic oral treatment improves spatial memory and motivated behaviors, reduces amyloid deposition and plaque-associated gliosis, and enhances hippocampal neurogenesis, supporting the proposed multi-target neuroprotective effects of NeuproGemp. Mechanistically, pentagalloylglucose (PGG) and tannic acid were identified as potent natural hQC inhibitors, providing a basis for reduced pyroglutamate-modified Aβ formation. These results highlight the translational potential of NeuproGemp as an early-stage intervention for Alzheimer’s disease. Future studies should evaluate its impact on tau pathology, directly measure *in vivo* hQC activity, assess long-term safety and efficacy, and optimize dosing regimens to support clinical translation.

## Data Availability

The raw data supporting the conclusions of this article will be made available by the authors, without undue reservation.
