# Supplementary material for: NeuproGemp, a polyphenol-rich botanical formula, ameliorates Alzheimer’s-like pathology in APP/PS1 mice via inhibition of human glutaminyl cyclase
Source: Front Pharmacol. 2025 Oct 21;16:1673532. doi: 10.3389/fphar.2025.1673532 (PMC12582971; doi:10.3389/fphar.2025.1673532)
Supplement: Supplementary file 1 [file Supplementaryfile1.docx]

**NeuproGemp, a Polyphenol-Rich Botanical Formula, Ameliorates Alzheimer’s-like Pathology in APP/PS1 Mice via Inhibition of Human Glutaminyl Cyclase**

**Tien-Sheng Tseng^1, 2*^, Chia-Ching Liaw^3^, Young-Ji Shiao^3^, Ya-I Huang^4^, Yu-Hsiu Cheng^4^, Wang-Chuan Chen^6, 7^, and Keng-Chang Tsai^3, 5*^**

^1^Institute of Molecular Biology, National Chung Hsing University, Taichung, Taiwan.

^2^Doctoral Program in Microbial Genomics, National Chung Hsing University and Academia Sinica, Taiwan.

^3^National Research Institute of Chinese Medicine, Ministry of Health and Welfare, Taipei, Taiwan.

^4^Department of Research and Development, Likang Biotechnical Co., Ltd., Tainan, Taiwan.

^5^Ph.D. Program in Medical Biotechnology, College of Medical Science and Technology, Taipei Medical University, Taipei, Taiwan.

^6^The School of Chinese Medicine for Post Baccalaureate, I-Shou University, Kaohsiung, Taiwan.

^7^Department of Chinese Medicine, E-Da Hospital, Kaohsiung, Taiwan.

*** Correspondence:**

Dr. Keng-Chang Tsai

tkc@nricm.edu.tw

Dr. Tien-Sheng Tseng

emersontseng@dragon.nchu.edu.tw

**Supporting information**


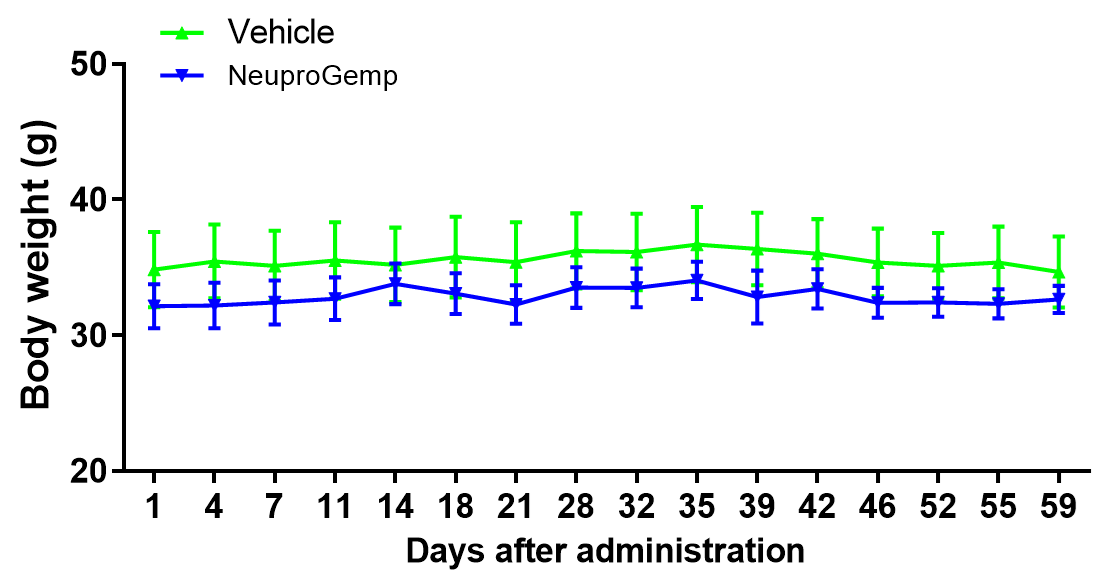
**Figure S1. Effect of NeuproGemp administration on body weight in mice.** Body weight was monitored in mice treated with vehicle (green line) or NeuproGemp (blue line) over a period of 59 days. Data are presented as mean ± SEM (n = 6 and 11 for Vehicle and NeuproGemp group, respectively). No significant changes in body weight were observed between groups throughout the experimental period, indicating that NeuproGemp administration did not cause overt systemic toxicity.


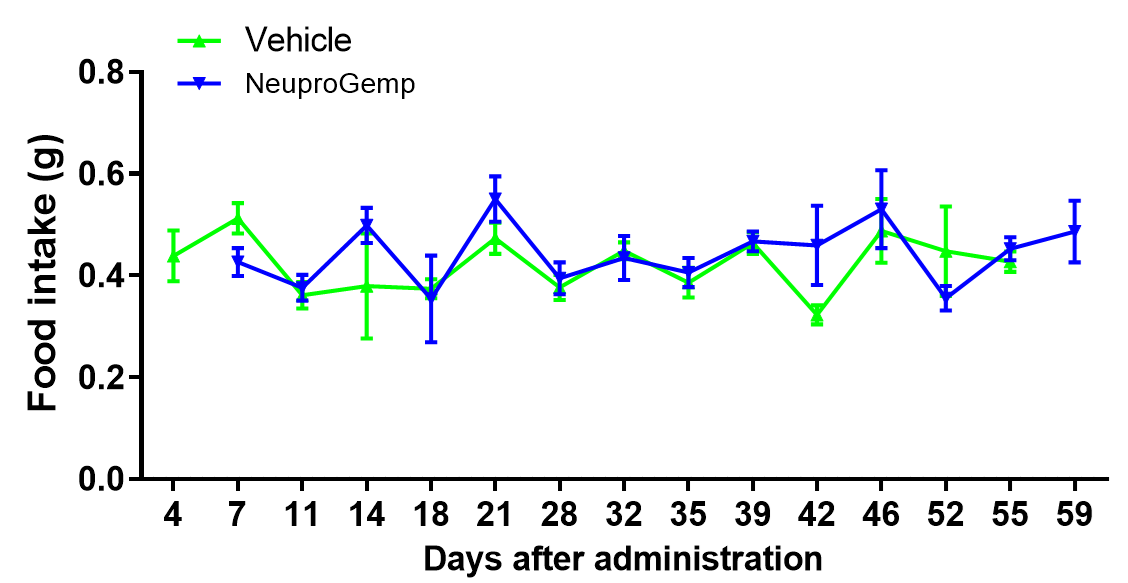
**Figure S2. Effect of NeuproGemp administration on food intake in experimental animals.** Food intake (g) was measured at the indicated time points over a 59-day period following administration of Vehicle (green triangles) or NeuproGemp (blue inverted triangles). Data are presented as mean ± SEM (n = 6 and 11 for Vehicle and NeuproGemp group, respectively). No significant differences in food intake were observed between groups throughout the study period, indicating that NeuproGemp treatment did not adversely affect feeding behavior.
